# Intra-Cardiac Flow from Geometry Prescribed Computational Fluid Dynamics: Comparison with Ultrasound Vector Flow Imaging

**DOI:** 10.1007/s13239-023-00666-2

**Published:** 2023-06-15

**Authors:** Rasmus Hvid, Matthias Bo Stuart, Jørgen Arendt Jensen, Marie Sand Traberg

**Affiliations:** Department of Health Technology, Technical University of Denmark, 2800 Kongens Lyngby, Denmark

**Keywords:** Intra-cardiac hemodynamics, Computational fluid dynamics, Geometry prescribed motion, Ultrasound blood flow imaging

## Abstract

**Purpose:**

This paper investigates the accuracy of blood flow velocities simulated from a geometry prescribed computational fluid dynamics (CFD) pipeline by applying it to a dynamic heart phantom. The CFD flow patterns are compared to a direct flow measurement by ultrasound vector flow imaging (VFI). The hypothesis is that the simulated velocity magnitudes are within one standard deviation of the measured velocities.

**Methods:**

The CFD pipeline uses computed tomography angiography (CTA) images with 20 volumes per cardiac cycle as geometry input. Fluid domain movement is prescribed from volumetric image registration using the CTA image data. Inlet and outlet conditions are defined by the experimental setup. VFI is systematically measured in parallel planes, and compared to the corresponding planes in the simulated time dependent three dimensional fluid velocity field.

**Results:**

The measured VFI and simulated CFD have similar flow patterns when compared qualitatively. A quantitative comparison of the velocity magnitude is also performed at specific regions of interest. These are evaluated at 11 non-overlapping time bins and compared by linear regression giving *R*^2^ = 0.809, SD = 0.060 m/s, intercept = − 0.039 m/s, and slope = 1.09. Excluding an outlier at the inlet, the correspondence between CFD and VFI improves to: *R*^2^ = 0.823, SD = 0.048 m/s, intercept = -0.030 m/s, and slope = 1.01.

**Conclusion:**

The direct comparison of flow patterns shows that the proposed CFD pipeline provide realistic flow patterns in a well-controlled experimental setup. The demanded accuracy is obtained close to the inlet and outlet, but not in locations far from these.

**Supplementary Information:**

The online version contains supplementary material available at 10.1007/s13239-023-00666-2.

## Introduction

Intra-cardiac blood flow patterns have been simulated since the 1970s by modelling simplified ventricle geometries [1, 21]. Since medical imaging and computational power have become more available, models and simulations of intra-cardiac blood flow patterns have been competing with direct measurement for obtaining patient-specific intra-cardiac blood flow patterns.

In recent years intra-cardiac blood flow patterns have been investigated as a potential clinical tool for diagnostics and decision-aid. Current methods are variations of ultrasound [6, 8, 9, 11] and cardiac magnetic resonance (CMR), which has several different sequences ranging from 2D phase contrast to the state-of-the-art 4D flow CMR [19, 24] are used. 4D flow CMR is considered state of the art for obtaining intra-cardiac blood flow patterns, but the method has the disadvantages of long acquisition times, averaging effects over multiple heart beats, and insufficient spatial and temporal resolution [24]. However, researchers have already correlated intra-cardiac blood flow patterns with pathology using flow-based metrics like vortex size and intensity [19] and changes in fractions between the flow types; direct flow, retained inflow, delayed ejection flow, and residual volume [5].

Computational fluid dynamics (CFD) models can estimate intra-cardiac blood flow patterns, and the metrics mentioned above can be derived from a simulation model as an alternative to direct measurement. Cardiac CFD models have advantages over direct measurement. In echocardiography current systems do not show the full velocity field in 3D with the 3D velocity vector, and, thus, do not reveal the whole picture of the ventricular flow. But in CFD models the data describing the full velocity field in 3D over time are available in any point at any time instant during the cardiac cycle. In 4D flow CMR imaging data are obtained by averaging up to several hundred heart beats, whereas the geometry for the presented CFD model is obtained from computed tomography angiography (CTA) where only a few cardiac cycles are required to reconstruct the geometry using a low radiation dose [26]. Furthermore, as previously mentioned, 4D flow CMR is limited by spatial and temporal flow resolution, where the resolution in both time and space of a simulation model is only limited by computer hardware and simulation time.

Simulation models are most effective and efficient when designed to a specific purpose, and therefore many modelling approaches have been developed for simulating intra-cardiac blood flow patterns. Doost et al. [4] presents an overview of common strategies for modelling cardiac flow. Choosing a strategy is based on data availability to feed the model and computational resources in terms of hardware and software. Since the heart phantom used in this study is compatible with ultrasound imaging and CTA, a dynamic patient-specific CFD model with geometry prescribed wall movement is chosen as the model approach. In addition, the formulation for handling the moving boundaries is based on the Arbitrary Lagrangian–Eulerian (ALE) formulation.

Validation of results achieved with i.e., in vivo patient-specific CFD simulations is difficult to perform for three main reasons. Firstly, what CFD simulations potentially offer is not always measurable using the current state-of-the-art methods. Second, the inter-measurement variability on the subjects blood flow patterns is confounded with the differences caused by the measurement method [27]. Lastly, acquiring in vivo data has limitations as described above. Despite its difficulties, in vivo studies have already been conducted by others, comparing patient-specific CFD simulations with the clinical state-of-the-art flow methods, reporting promising results [16, 17]. Lantz et al. [16] compares metrics such as kinetic energy, stroke volume, etc. While the study shows correlation on important metrics, the data are still limited by inter-measurement variability and measurement limitations. The dynamic heart phantom setup utilised here allows for controlled and systematic high quality ultrasound measurements based on vector flow imaging (VFI) [15, 20] with precisely defined spatial relationships in a repeatable setup. VFI provides 2D vector velocity fields acquired in real-time, where quantitative data can be extracted from everywhere in the image. Therefore, it is ideal for comparison to simulated flow data in the heart as performed here.

This paper describes and applies a geometry prescribed CFD pipeline on a dynamic heart phantom with the purpose of validating the flow dynamics obtained from the pipeline.

It is hypothesised that the results of the CFD pipeline proposed here, i.e., the flow patterns and quantities, can accurately model the physical intra-ventricular flow. The model is considered accurate if the computed flow is within one standard deviation of the measured flow, i.e., the experimental VFI reference. This would show that the output of the pipeline is reliable and that the pipeline can be applied to in vivo cases.

The paper is organised as follows. In “Methods” section a complete description of the applied methods is provided. It includes the individual elements of the pipeline starting out with the dynamic heart phantom, then the data acquisition with CTA and VFI is presented followed by a description of the CFD simulation details. Hereafter, the results are presented in “Results” section and discussed in “Discussion” section. The paper is concluded in “Conclusion” section.

## Methods

**Fig. 1:**
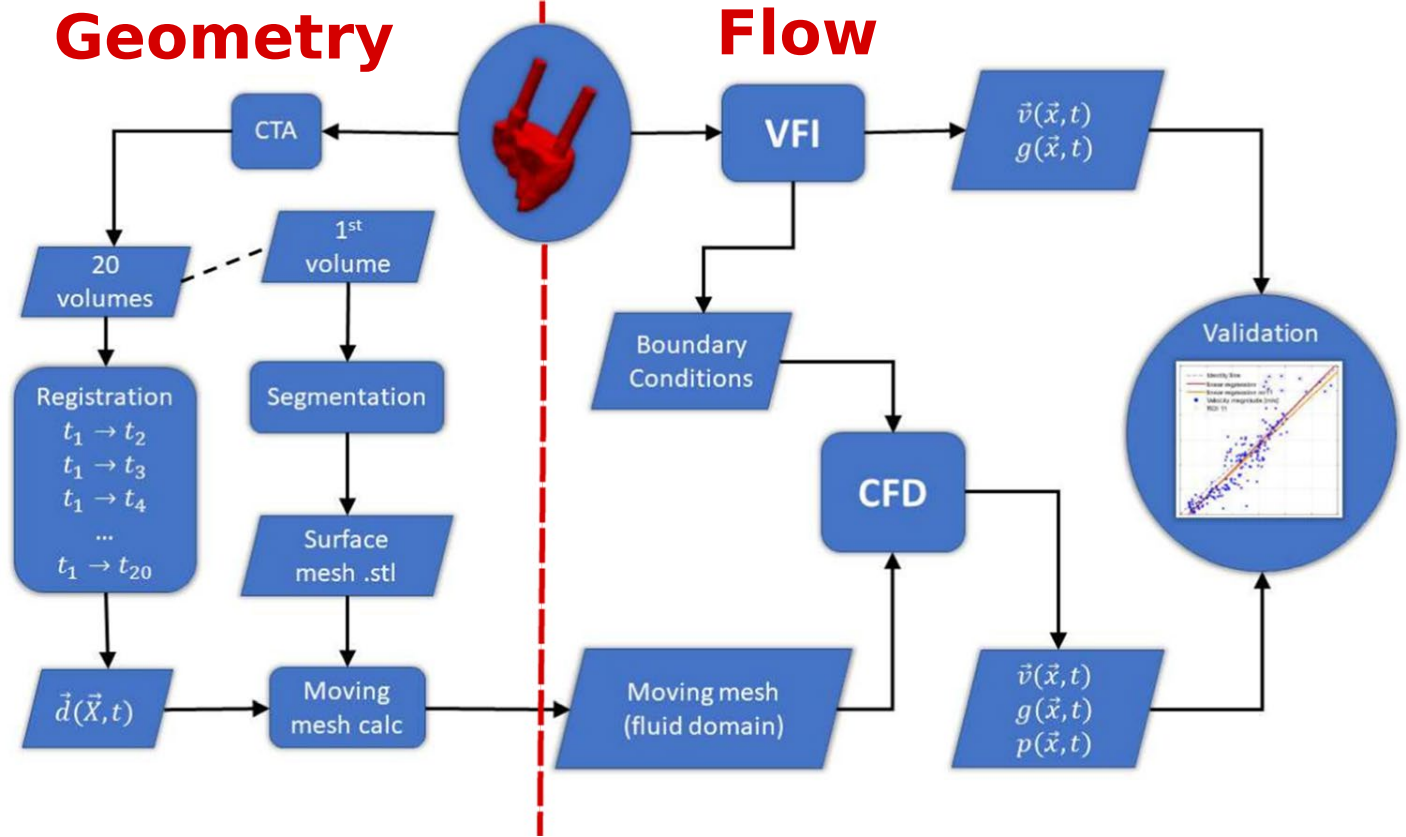
Method flowchart for the CFD pipeline, where the part to the left of the red dashed line concerns the geometry, and the part to the right of the red dashed line concerns the flow simulation. The center is the lumen of the heart phantom visualised in red. The phantom is imaged by CTA and VFI to obtain boundary conditions for the CFD simulation and velocity data for validation. Rounded corners on the boxes represent methods of data processing. Sharp corners represent data sets and the circles indicates input (the heart phantom) and output (result of the validation). In the flowchart $g(\vec{x},t)$ represent the geometry, here the B-mode for the VFI and domain index for the CFD. The motion obtained from registration is labelled $\vec{d}(\vec{X},t)$, and $\vec{v}(\vec{x},t)$ is the velocity field, $p(\vec{x},t)$ represents the pressure field

An overview of the CFD pipeline is presented as a flow chart in Fig. 1. The following presents the individual elements of the CFD pipeline and how the information is used to obtain the results.

### Dynamic Heart Phantom

**Fig. 2:**
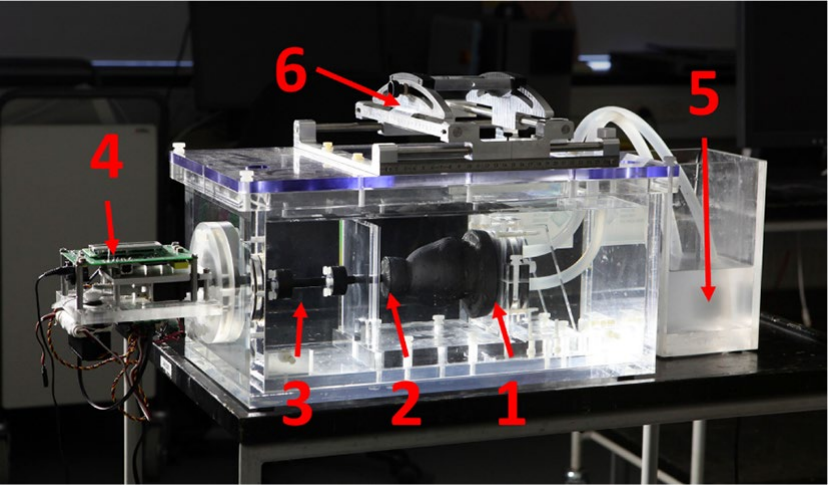
Photograph of the DHP [12]. **1**: Base of the heart phantom with inlet and outlet tubing. **2**: Apex of the DHP. **3**: Actuator rod for transfer of motion. **4**: Servo motors and micro-controller to apply the motion. **5**: Fluid reservoir. **6**: Fixture for the ultrasound probe where angle and position is adjusted

The CFD pipeline is applied to a dynamic bi-ventricular heart phantom (DHP) from Shelley Medical Imaging Technologies (Toronto, Canada). The DHP wall surrounding the two ventricles is made of polyvinyl alcohol with additives, and the phantom has no heart valves. The additives make the ventricle walls appear similar to the myocardium on computed tomography images and during ultrasound scans. The DHP is submerged into a water-filled tank and anchored at the base, see Fig. 2.

**Fig. 3:**
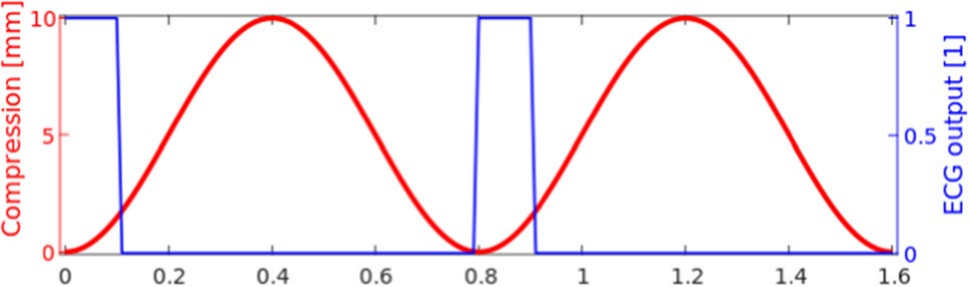
Movement as prescribed to the micro-controller and applied to the phantom. Red: Compression applied to the actuator rod. Blue: The ECG output for time synchronisation in CTA

An actuator rod is attached to the apex of the heart phantom, and two servo motors are attached to the actuator rod, one pushing the rod introducing a compression and one rotating the rod introducing a torsional movement of the heart phantom. The servo movement is programmed on a micro-controller, which has three default output channels: compression, torsion and electrocardiography (ECG) output. The movement prescribed to the DHP here is a pure compression of 10 mm along the long-axis without rotation. This is a simplification of the in vivo cardiac motion where both compression and rotation are seen. The ECG output facilitates synchronisation in time, see Fig. 3, and is used for time synchronisation with the CTA. In this study only the right ventricle (RV) is measured and simulated.

In Fig. 3 two flow cycles are visualised, however in the experimental setup the movement runs for several hundred cycles to ensure periodic flow patterns independent of initial conditions at the time of measurement. A micro-controller was programmed to provide motor inputs to move the actuator rod according to:

1 $$\begin{aligned} c(t) = C_{\rm d} \sin ^2 \left( \frac{\pi \cdot t}{0.8\,\text {s}}\right) , \end{aligned}$$

which corresponds to a compression of $C_{\rm d}=10$ mm every 0.8 s, i.e., 75 beats per minute. The actual flow cycle period was measured to be 0.76 s in both CTA and ultrasound measurements. The reason for this discrepancy is unknown. Since the two different measurements agree, a cycle period 0.76 s is assumed in the VFI processing and in the CFD model, as this represents the real conditions most accurately.

During the gathering of imaging data and flow measurements, blood mimicking fluid [23] is circulated through the ventricle inlet and out of the outlet via tubes from a fluid reservoir, see Fig. 2. The flow is governed by an impeller pump (Aqua Nova NBF-300, Aqua Nova, Poland) submerged into the fluid reservoir. The pump provides a constant flow rate, which is quantified in the phantom inlet using VFI, see “Computational Fluid Dynamics” section. The peak volume flow rate at the RV inlet is 2.21 L/min. The total fluid volume in the phantom ventricle, tubes and reservoir is approximately 700 mL. This fluid has a density of $\rho$ = 1037 ± 2 kg/m$^3$, and a viscosity of $\mu$ = 4.1 ± 0.1 mPa s [23]. Further, it behaves as a Newtonian fluid in the applied experimental conditions.

### Computed Tomography Angiography

The dynamic heart phantom was scanned in a commercial CT scanner (Toshiba Aquilion ONE, Canon Medical Imaging Systems Inc., Japan) applying a CTA protocol while undergoing the exact displacement described in “Dynamic Heart Phantom” section. The CTA was timed using the simulated ECG output, which is synchronised with the phantom movement as visualised in Fig. 3. The CTA output data consist of 20 volumes, i.e., 5% phase increments from peak to peak in the ECG. To ensure synchronisation between the CTA volumes and the CFD, each volume was compared to the corresponding geometry at these times in the CFD simulation. The voxel size in the reconstruction is $0.6 \times 0.6 \times 0.5$ mm^3^. The heart cycle period was measured to be 0.76 s resulting in a volume-sample period of 0.038 s. The CTA procedure is the same as for an in vivo measurement with ECG electrodes connected to the DHP ECG output channel, and intravenous contrast fluid was added to the fluid reservoir to obtain proper contrast between the fluid and the solid wall material similar to in vivo CTA.

### Vector Flow Imaging

**Fig. 4:**
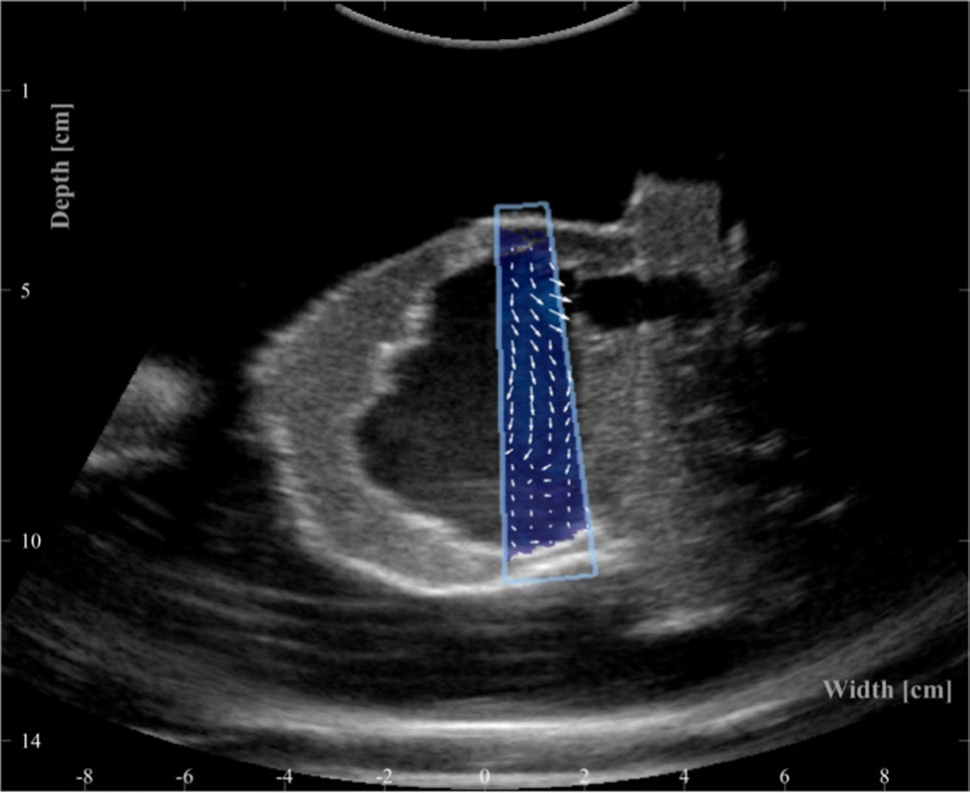
Example of a full B-mode image with VFI in a sub-region. The arrows direction and length indicate flow direction and magnitude

**Fig. 5:**
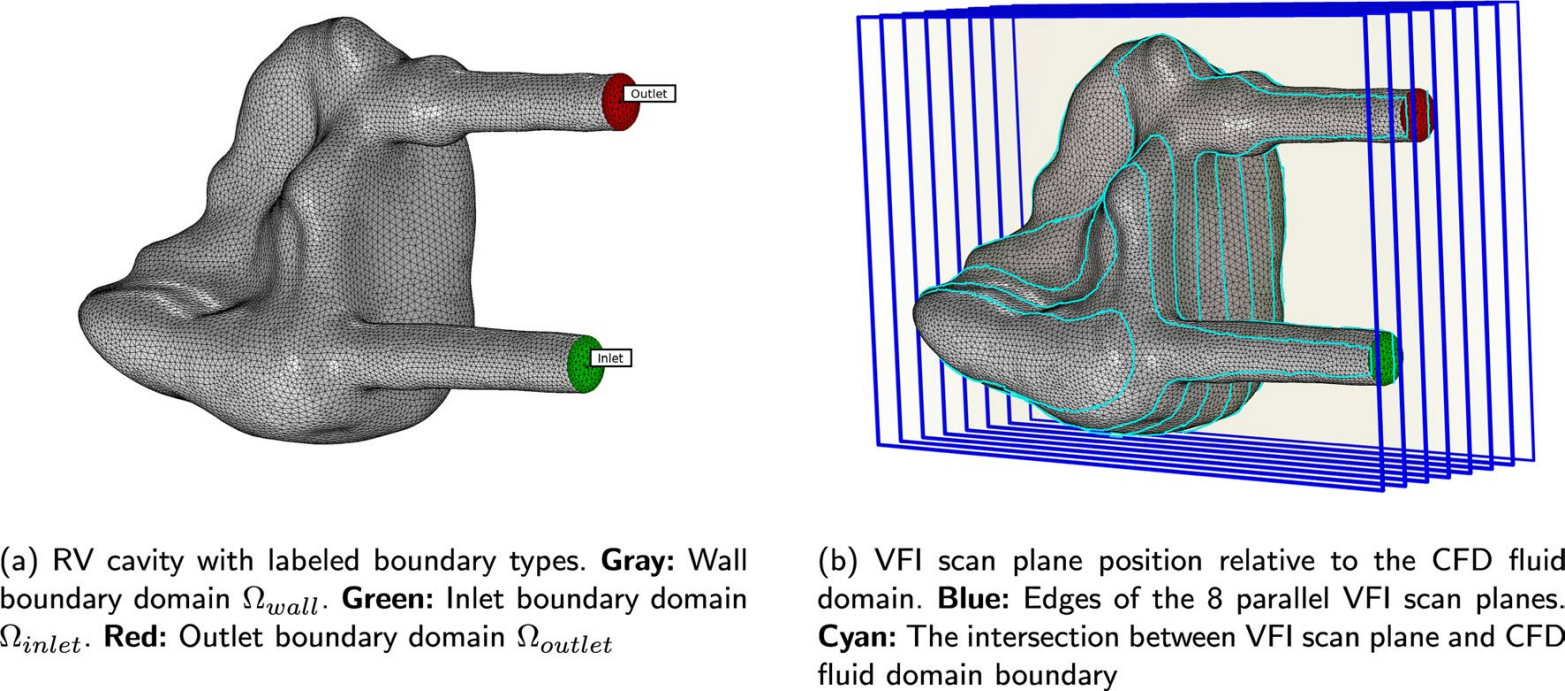
Visualisation of the segmented RV from CTA at the initial configuration, i.e., $t = 0$ s

In-plane time dependent velocity fields were measured on the DHP with ultrasound VFI using a modified scanner for research (BK5000, BK Medical ApS, Herlev, Denmark) and a convex ultrasound probe (6C2,BK Medical ApS, Herlev, Denmark). The applied ultrasound sequence estimates the time dependent in-plane blood velocity vector field [14, 15] with a resolution of $0.75 \times 1.5$ mm given that the wavelength of the ultrasound wave is 0.375 mm. During VFI data acquisition the B-mode image is used to navigate the anatomy. The in-plane velocity magnitudes and directions are measured and visualised inside a sub-region that the operator draws on top of the B-mode image during acquisition as illustrated in Fig. 4. The narrow colour box is necessary to ensure sufficiently high sampling rate for the VFI data to capture the flow dynamics. The acquisition is time dependent, and can be saved as a video or as a raw data file. The probe was mounted on an in-house fixture on top of the DHP box, see Fig. 2, that enables full control of the probe position and tilt relative to the heart phantom. The fixture is used to scan eight parallel planes with an inter-plane distance of 5 mm as seen in Fig. 5.

In each plane several acquisitions are made for a duration of 6 to 8 s, corresponding to seven to ten cardiac cycles. Each VFI acquisition contains a full width B-mode image and VFI data inside a narrower sub-region of the B-mode image as seen in Fig. 4. The post-processed VFI data used for comparison contain data from 42 separate VFI acquisitions; 8 planes of 4–6 separate VFI acquisitions per plane, 210 cardiac cycles in total. The ultrasound equipment did not have ECG triggering to synchronise the cardiac phase of the measured cardiac cycles. Therefore, to ensure that all 42 VFI acquisitions are in phase, a time-delay was defined for each acquisition and added to the time stamps. The time-delays were estimated using a cross correlation of the B-mode images on a registration based displacement metric, i.e., by the movement of the phantom observed in the B-mode images.

### Computational Fluid Dynamics

**Fig. 6:**
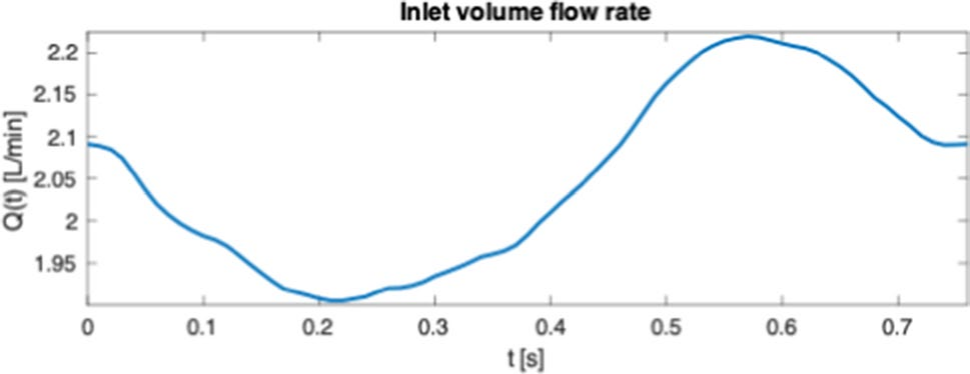
The time dependent inlet volume flow rate *Q*(*t*) applied to the inlet boundary as a plug flow. *Q*(*t*) was obtained by a VFI measurement and an estimate of the cross sectional area at the VFI measurement

The simulation pipeline is based on the geometry prescribed CFD, which applies a known movement to the boundary of a fluid domain [4]. How the fluid moves inside the fluid domain is determined by the boundary conditions applied to the three boundaries seen in Fig. 5, and defined as

2 $$\begin{aligned} & \Omega _{\rm inlet}: \vec{U}_{\rm inlet}(t)\\ &\Omega _{\rm outlet}: 0 \quad \text{ Pa } \\ & \Omega _{\rm wall}: \vec{U}_{\rm mesh}(t) \quad ,\\ \end{aligned}$$

where the boundary condition at the inlet, $\Omega _{\rm inlet}$, is a time dependent plug flow prescribed by a mean velocity magnitude $\vert \vec{U}_{\rm inlet}(t)\vert$. The velocity magnitude is estimated from the inlet cross-section area in the geometry representing the simulation model and demanding the same inlet volume flow rate *Q*(*t*) as in the VFI measurement. *Q*(*t*) is obtained from the VFI data by

3 $$\begin{aligned} Q(t) = \vert \vec{U}_{\rm inlet}(t)\vert \cdot (\pi (D/2)^{2}) \quad , \end{aligned}$$

where $\vert \vec{U}_{\rm inlet}(t)\vert$ is acquired along the diameter *D* of the inlet assuming that the inlet is circular. This quantification of *Q*(*t*) is necessary because the position of the inlet in the simulation model is different from the position of the VFI measurement. The inlet in the simulation model is defined upstream in the entrance tube, see Fig. 5, compared to where the inlet velocity is measured with VFI. In the simulation, this is done to allow the flow profile to develop prior to entering the RV. Because none of the VFI scan planes line up with the inlet tube diameter, see Fig. 5, velocity data were extracted from the plane, which covers the majority of the inlet diameter. Specifically, the velocity field along a line perpendicular to the flow at the transition between the inlet tube and the RV volume is extracted, and the spatial mean velocity is calculated for each time step throughout the flow cycle. Due to this offset of the imaging plane, this measured mean velocity is estimated to be 80% of the true mean velocity under the condition that the flow profile is parabolic. When compensating for the underestimation of the true spatial mean velocity the difference amounts to 0.386 m/s at $t = 0$ s. This is added to the applied inlet velocity waveform to obtain the same volumetric flow rate at the inlet in the CFD model as in the VFI measurement. The volume flow rate acquired from VFI is plotted in Fig. 6. The inlet velocity profile, $\vec{U}_{\rm inlet}(t)$, is prescribed as a plug flow, but in reality the spatial velocity profile deviates from this to a more blunt parabolic-like profile at the RV entrance. This is obtained by allowing the profile to develop in the inlet pipe prior to entering the RV volume.

The boundary condition at the outlet, $\Omega _{\rm outlet}$, is a constant pressure of 0 Pa, which corresponds to the outlet tube going into a reservoir with a constant fluid column height. The boundary condition at the wall, $\Omega _{\rm wall}$, is the no-slip condition. In this model no-slip is defined as the fluid velocity being equal to the mesh movement velocity at the wall boundary. The CFD is solved numerically in COMSOL Multiphysics v5.6 (COMSOL AB Stockholm, Sweden) using the finite element method and the ALE formulation. Here the Navier–Stokes equations for an in-compressible fluid,

4 $$\begin{aligned} \rho \left( \frac{\partial \vec{U}(\vec{r},t)}{\partial t} + (\vec{U}(\vec{r},t)\cdot \nabla )\vec{U}(\vec{r},t) \right) \nonumber \\ = -\nabla p + \mu \nabla ^2\vec{U}(\vec{r},t)&\end{aligned}$$

5 $$\begin{aligned} \nabla \cdot \vec{U}(\vec{r},t) = 0 \end{aligned}$$

are solved numerically on a moving mesh assuming isothermal laminar flow. In (4) and (5) $\vec{U}(\vec{r},t)$ is the fluid velocity vector (m/s) which is depending on both time *t* (s) and space $\vec{r}$ (m). Here $\vec{r} = x \hat{e}_{x} + y \hat{e}_{y} + z \hat{e}_{z}$ is the position vector for a given point (*x*, *y*, *z*) in the fluid domain. Further, *p* is pressure (Pa), $\rho$ is the fluid density (kg/m^3^), and $\mu$ is the fluid viscosity (Pa s). $\nabla$ is the del operator, and $\nabla ^2$ is the Laplace operator. The effects of gravity are negligible, and gravity is therefore ignored. The fluid density and viscosity in the presented CFD model are assigned those of the blood mimicking fluid used in the experimental measurements with CTA and VFI (see “Dynamic Heart Phantom” section.

#### Geometry

The fluid domain is defined as a surface mesh obtained from segmentation of the right ventricle (RV) in a single CTA volume (see “Computed Tomography Angiography” section) at the first time instance, which corresponds to minimum compression in the presented configuration. The segmentation is performed in MATLAB (Mathworks Inc., Natick, MA) and the final geometry is visualised in Fig. 5. The full extend of the inlet and outlet tubes is kept as part of the segmentation to ensure smooth transition between the moving outer wall of the RV volume and the walls of the inlet and outlet pipes, which are not moving.

The ventricle movement is prescribed to the surface mesh via a displacement field, which is obtained by volumetric image registration. See “Registration” section.

#### Registration

The ventricle movement is estimated by volumetric image registration of the 20 CTA volumes. Several separate registrations are performed as illustrated in Fig. 1. These registrations are between the first volume and every other volume in the CTA sequence. At $t=0$ s the displacement is naturally zero. Furthermore, since the movement is cyclic, zero displacement is appended to the temporal displacement field, resulting in 21 time instances. The resulting displacement field contains a displacement vector component in each direction (*x*, *y*, *z*) in each voxel at each time step. The resulting displacement field is represented by the displacement field $\vec{d}(x, y, z, t)$

6 $$\vec{d}(x, y, z, t)= \left[\begin{array}{l} d_x(x,y,z, t) \\ d_y(x,y,z, t) \\ d_z(x,y,z, t) \end{array}\right]$$

The registration uses the MATLAB function imregdemons, which is based on the Diffeomorphic Demons algorithm [28, 29]. Finally the displacement is smoothed temporally, interpolated in MATLAB and sent to the COMSOL model solver during the mesh movement computations.

#### CFD Steps in COMSOL

The numerical computation can be split into two independent steps to decrease complexity; The mesh movement (pure Lagrangian), and the CFD (ALE on the moving mesh nodes from the mesh movement step). This split allows the movement to be calculated separately, saving computation time by allowing larger time steps, and avoiding re-calculating the mesh positions for every cycle by reusing the cyclic mesh movement solution for each cardiac cycle.

Mesh Movement The mesh movement is obtained by applying the discrete displacement field, $\vec{d}(x,y,z,t)$, on the surface mesh of the fluid domain. First, the displacement field is interpolated and smoothed temporally. The temporal smoothing is penalised to ensure equal rates of volume change at the beginning and end of the cardiac cycle. The displacement is applied directly to the fluid domain surface. The movement of the volume inside the fluid domain is determined by a hyper-elastic spatial smoothing. The output of this study step is a moving mesh of one cardiac cycle, where every boundary node has the exact same position in the beginning, and in the end of the cardiac cycle. The mesh positions are calculated at fixed time steps of 0.019 s. The mesh movement solution is stored and used as input for the CFD solver, which interpolates the solution for each time step taken by the CFD solver.

Ensuring Well-Posed Initial Conditions A stationary solution is calculated and used as initial condition to avoid solving an ill-posed model. A series of stationary solutions are calculated at $t = 0$ s on the initial reference geometry with zero displacement with increasing inlet velocity from 0 m/s to the inlet velocity at $t = 0$ s. Each stationary solution uses the previous solution as an initial guess. The final stationary solution is used as the initial condition for the first cycle of the dynamic solution.

**Table 1:** Time dependent solution: key numbers and resources

Total elements	384,054
Degrees of freedom	391,324 (plus 384,055 internal DOFs)
Computation time	16 h 38 m 33 s
Resources	2× Intel Xeon CPU E5-2660 v3
	2 sockets, 20 cores, 2.60 GHz
	Available memory: 128.65 GB (utilized memory): 33.94 GB

**Fig. 7:**
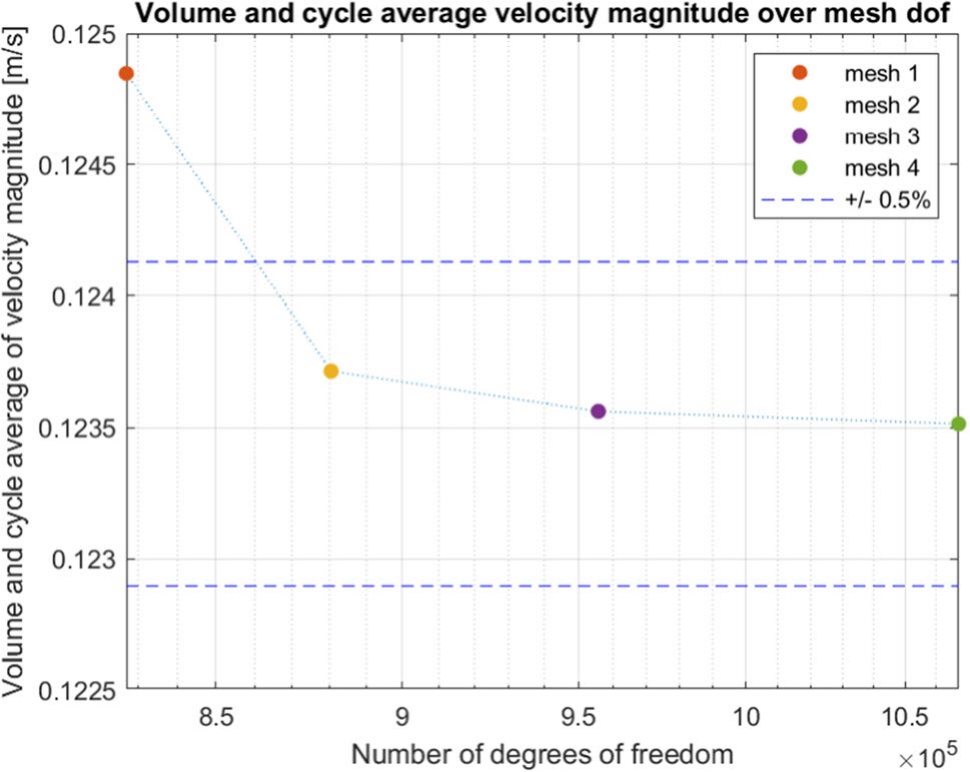
Mesh independence study. The mesh size is provided in degrees of freedom on the *x*-axis. The meshes are colour coded and the dashed horizontal lines mark a difference of 0.5% compared to the mesh with the highest number of degrees of freedom

Mesh Independence Study A mesh independence study was performed to check the quality of the mesh and the numerical accuracy of the simulation results. Four different meshes were tested on the model geometry and the metric on which the meshes were evaluated is the volume and cycle average of the velocity magnitude. The result is shown i Fig. 7. The mesh independence study indicate that the solution is precise within 0.5% when the number of degrees of freedom is larger than $8.8 \times 10^{5}$. For this study the settings in Mesh 4 were used.

Please note that the degrees of freedom listed in Tab 1 is calculated differently compared to the degrees of freedom listed in Fig. 7, which is based on all compiled equations in the simulation.

**Fig. 8:**
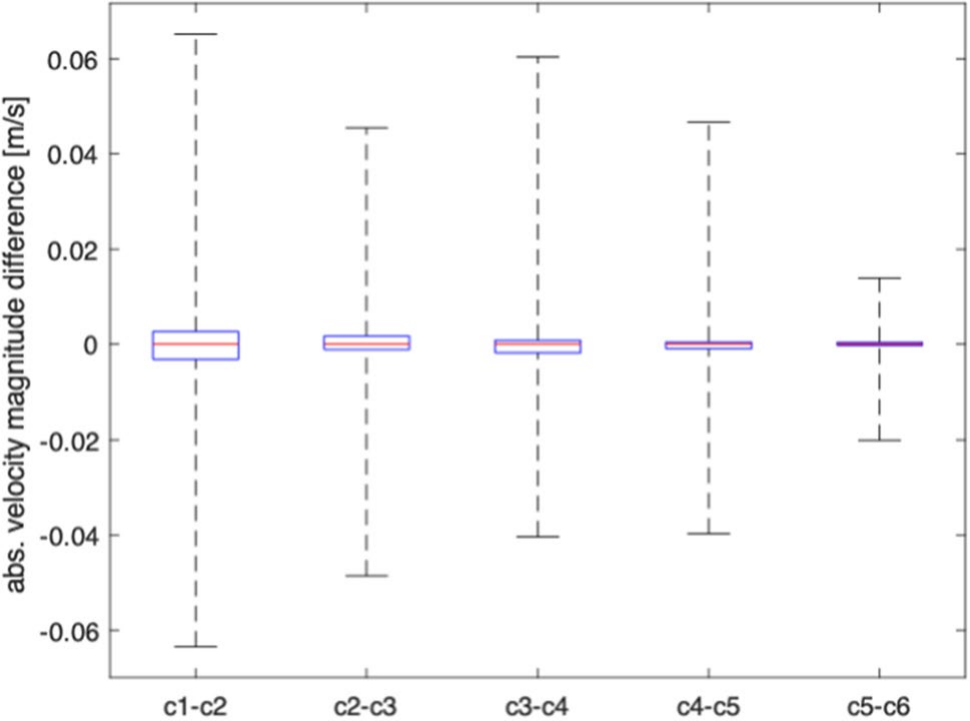
Cycle convergence evaluation. Each box plot visualises difference in velocity magnitude between consecutive cycles measured in 97,831 points

Time Dependent CFD The mesh movement solution calculated in the first step is applied to all mesh nodes using the ALE formulation. The boundary conditions are defined in (2). The simulation is run for six cycles to ensure cycle convergence, and thereby independence from initial conditions. The convergence is evaluated by calculating the difference in velocity magnitude at evenly distributed volumes in the fluid domain at the last time step of each cycle, such that

7 $$Vel_{\text{diff}} = \vert \vec{U}_{i} \vert - \vert \vec{U}_{i+1} \vert$$

where $Vel_{\text{diff}}$ is the quantified difference in velocity magnitude in a volume, and *i* is the flow cycle index running from one through five. The result of the convergence is given in Fig. 8. It is seen that the absolute difference in velocity magnitude is reduced consecutively as more cycles are run. The difference in absolute velocity magnitude when comparing cycle 1 to cycle 2 is 0.133 m/s, whereas when looking at this difference between cycle 5 and 6, it is reduced to 0.038 m/s. The CFD simulations were run on a computer with Intel Xeon CPUs with details on resources provided in Table 1. The computation time was approximately 17 h.

### Post Processing

**Fig. 9:**
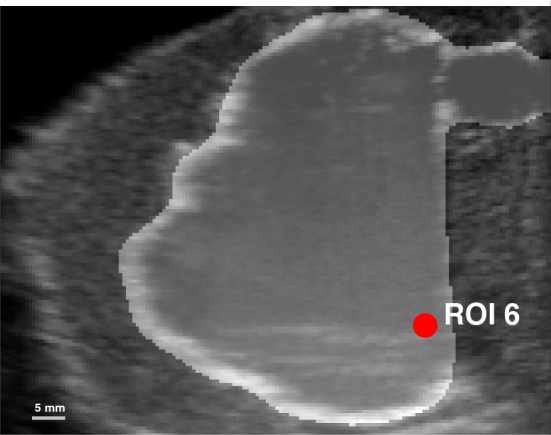
Illustration of the fluid domain layered on top of the B-mode image. In this plane ROI #6 is seen at the base of the heart phantom

The CFD simulation solution contains a time dependent 3D flow velocity field and pressure field, as well as domain (geometry) information. The VFI is limited to eight parallel planes containing time dependent in-plane 2D flow velocities, and the corresponding time dependent B-mode images as the geometry information. For direct comparison the two data sets are interpolated onto the same spatial-temporal grid consisting of eight planes with a pixel size of $0.5 \times 0.5$ mm^2^, and a temporal sample period of 0.01 s. The information available for each pixel at each time point in each plane is therefore: *u*, *v*, *w*, and *g*, where *u*, *v*, and *w* are the velocity vector components along each direction in the global coordinate system, such that

8 $$\vec{U}(x,y,z,t) = \left[ {\begin{array}{*{20}l} {u(x,y,z,t)} \hfill \\ {v(x,y,z,t)} \hfill \\ {w(x,y,z,t)} \hfill \\ \end{array} } \right]\begin{array}{*{20}l} {x{\text{ - comp}}{\text{.}}} \hfill \\ {y{\text{ - comp}}{\text{.}}} \hfill \\ {z{\text{ - comp}}{\text{.}}} \hfill \\ \end{array}$$

and *g* is geometry, i.e., B-mode for the ultrasound and domain index for the CFD. The geometry is used for co-registration of the two data sets by aligning landmarks using a rigid transformation. In Fig. 9 the CFD fluid domain is visible as a transparent grey overlay on top of the B-mode image.

Note that the CFD data contains more information than the VFI data, because the VFI does not have any pressure information and only measures in-plane 2D velocities. The *v* component is, in this situation, the out-of-plane velocity vector component of the VFI and therefore not included in the analysis. Hence, the comparisons are only made from in-plane velocities, and the *v* component of the CFD is not included either in the comparisons made here. The magnitude of the out-of-plane component from the CFD data is provided in the results to illustrate the importance of having the full velocity information when analysing complex flow as in the heart.

### Method and Metrics for Comparison

The qualitative comparison is a description of flow trends in corresponding VFI slices and CFD slices. Thus, it is based on visual assessment of flow direction and magnitude at two specific time points during the flow cycle. The first time instant is at 0.34 s corresponding to 45% into the flow cycle. The second time instant is at 0.74 s, which corresponds to 97% of the total flow cycle. The metric for quantitative comparison is the velocity magnitude,

9 $$\begin{aligned} \vert \vec{U} \vert = \sqrt{u^{2} + w^{2}} \end{aligned}$$

where *u* and *w* are the components of the velocity vector $\vec{U}$ as defined in (8) and $\vec{U}$ is evaluated at all time points in 16 regions of interest (ROI) distributed over the eight planes. The location of the 16 ROIs was selected manually with the aim to represent physiologically meaningful landmarks such as the inlet jet, the apex and the outlet tract. For the results presented here four specific ROIs where selected among the 16 because they represent some common findings. They are; at the inlet jet, close to the outlet tract, at the apex close to the moving wall, and in the almost cone shaped part of the RV close to the moving wall. The position of all 16 ROIs can be found in Online Resource 2. Note that the ROIs are stationary in space through out the flow cycle even though the fluid domain is moving. The velocity magnitude data for all ROIs are grouped in eleven non-overlapping time bins and averaged within each ROI. In both qualitative and quantitative comparisons, the VFI data are an average over five cycles. The CFD data are obtained from the last flow cycle in the simulation.

## Results

The result section is divided into a qualitative comparison of the simulated and measured velocity field in representative 2D planes, followed by a quantitative comparison of the flow in representative ROI’s through out the cardiac cycle.

### Velocity Field Comparison

**Fig. 10:**
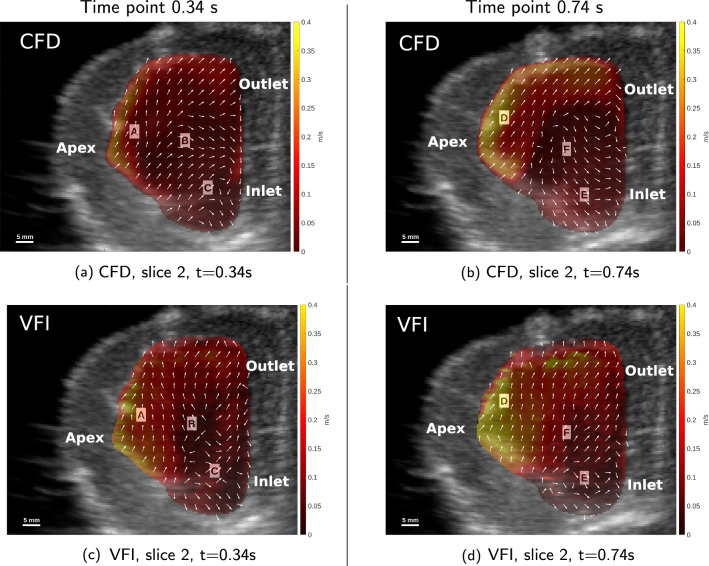
Comparative visualisations of in-plane velocities in slice 2 at the time-points $t = 0.34$ s (left column) and $t = 0.74$ s (right column). All visualisations are on top of a B-mode image at the corresponding time-point. The colour represents in-plane velocity magnitude inside the fluid domain, the arrows are normalised in-plane velocity directions. **a**, **b** CFD in-plane velocities. **c**, **d** VFI in-plane velocities

**Fig. 11:**
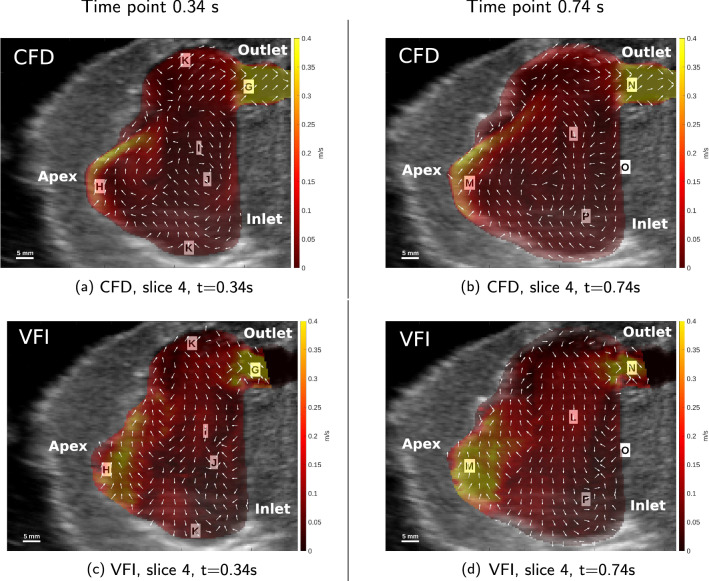
Comparative visualisations of in-plane velocities in slice 4 at the time-points $t=0.34$ s (left column) and $t=0.74$ s (right column). All visualisations are on top of a B-mode image at the corresponding time-point. The colour represents in-plane velocity magnitude inside the fluid domain, the arrows are normalised in-plane velocity directions. **a**, **b** CFD in-plane velocities. **c**, **d**: VFI in-plane velocities

In Fig. 10 the in-plane velocities for CFD and VFI at $t = 0.34$ s in two slices are shown, and in Fig. 11 the same shown is show at $t = 0.74$ s. These two slices and time instances are chosen because they showcase both good as well as poor correspondence in the velocity fields. The dynamics for all CFD slices and all VFI slices are available as additional data (Online Resource 1) visualising the measured and simulated flow for the complete flow cycle defined in this study. The colour bar indicates the velocity magnitude, and the arrows show the flow direction in each slice. Each image is also labelled with three anatomical landmarks on the phantom; the inlet, the outlet and the apex. In Fig. 10a and c the velocity vectors in both the CFD and VFI data show a general clockwise flow trend, especially to the left indicated by the label A, and low velocity (< 0.05 m/s) in the central region at label B.

The flow in the lower part at label C is down towards the right corner for both CFD and VFI. Additionally, a clockwise vortex to the right of label C is seen in both cases. In the domain centre, at label B, a vortex is observed together with very low velocities (<0.05 m/s) in the VFI data. In the simulated flow there is also rotation at label B, but it is not as pronounced as in the measured data when looking at the arrow directions and magnitudes.

At the end of the flow cycle at $t = 0.74$ s, visualised in Fig. 10b and d, both CFD and VFI have a clockwise rotational flow, with a dominant north-east flow direction in both at label D. In the bottom of the fluid domain, at label E, the direction is downwards and flow separation is seen, especially in the VFI data. At label F, opposite flow directions in CFD and VFI are observed, however, at the boundary to the right of label F, the flow is in the same direction for both techniques. There is a clockwise vortex in the simulated flow just above label F, and a similar vortex is seen in the VFI at label E in Fig. 10d.

In Fig. 11a and c the highest velocity of 0.4 m/s in both cases is through the outlet at label G. In both CFD and VFI there is an upwards directional flow at label H, and a downwards directional flow at label I, creating a clockwise vortex between label I and label H. A second clockwise vortex is seen at label J for both cases. In both CFD and VFI there is a flow separation between fluid going through the outlet towards the right, and going downwards below the outlet. In the bottom and top of the domain, both labelled K, there is a downward trend of the flow in the CFD, but the opposite is seen in the VFI.

Lastly, in Fig. 11b and d, the flow trend towards south in the centre above and below label L, additionally an upwards flow trend is seen at label M at the apex. Note here, that the apex is not moving at this time instant. In both CFD and VFI the highest velocity is at label N in the centre of the outlet (> 0.35 m/s) and the flow direction is downwards at the boundary to the right, labelled O, which corresponds to the base of the heart phantom. Besides high velocity at the outlet, the velocity is also high at the apex of the heart phantom at label M in both cases. Further, there is a vortex at label P in both situations, however, the vortex is clockwise in the simulated flow and counterclockwise in the measured flow. A clockwise vortex is seen in the CFD data between label M and label P, where the same clockwise vortex in VFI is located between label M and label L, and is not as pronounced as in the simulated flow.

Regarding the velocity magnitude, in general, the VFI data show slightly higher velocity when comparing the distribution of the dynamic range of the colour bar. For example, in Fig. 10a and c this is seen at the apex near label A. To obtain a more specific assessment of the velocity magnitude, it is obtained in four ROIs (see “Quantitative Assessment of the Flow).

Overall the qualitative assessment of the intra-cardiac flow patterns in measurement and simulation shows similar trends in most cases, when comparing CFD to VFI.

### Quantitative Assessment of the Flow

**Fig. 12:**
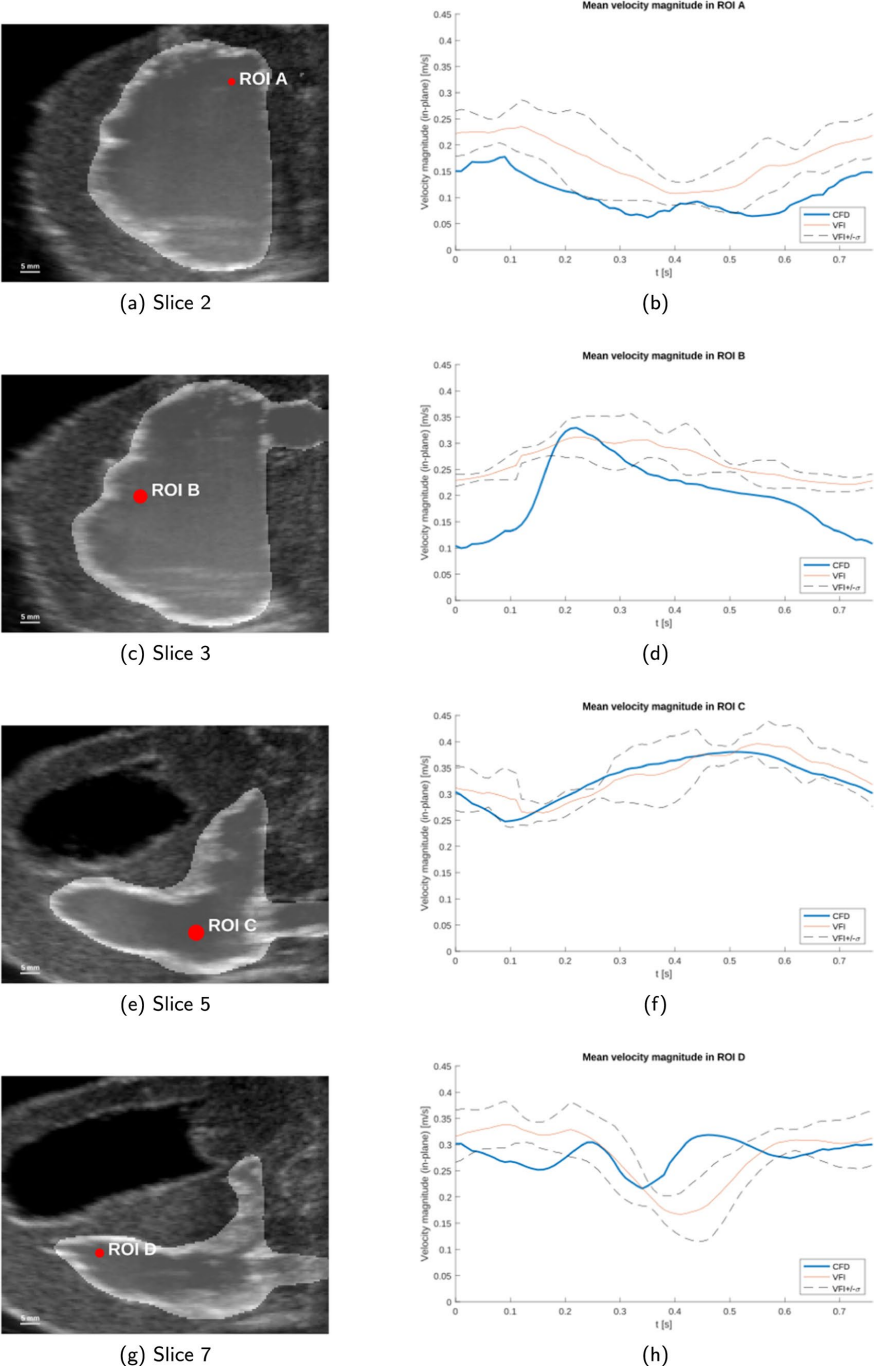
ROI placement and temporal velocity magnitude data for ROIs: A, B, C and D. Left **a**, **c**, **e**, **g** B-mode images with CFD fluid domain overlay, all taken at $t=0$ s. The red dot represents the location of the ROI. Right **b**, **d**, **f**, **h** The mean velocity magnitude over time inside each ROI for the CFD simulation and VFI measurement. The VFI flow profiles are averaged over five flow cycles. Systolic phase (compression) is from 0 s to 0.4 s, diastolic phase is from 0.4 to 0.76 s

**Fig. 13:**
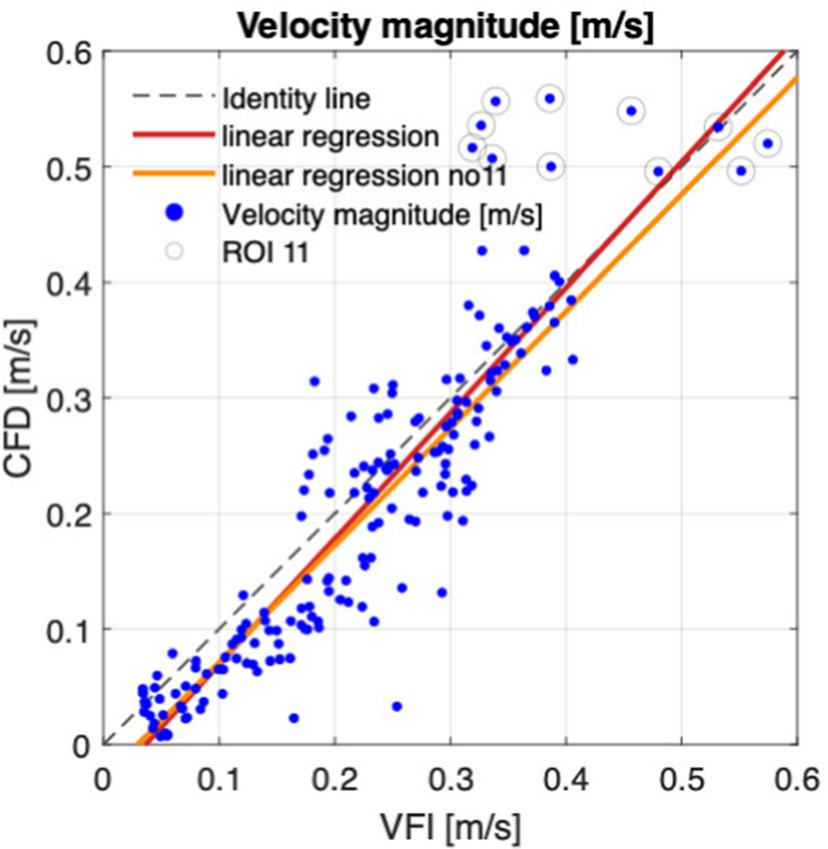
Comparison of velocity magnitude in 11 time bins at all 16 ROIs. *Red line* Linear regression on all data points ($R^2$ = 0.809, intercept = − 0.039 m/s, slope = 1.09, SD = 0.060 m/s). *Orange line* Linear regression excluding data from ROI #11 ($R^2$ = 0.823, intercept = − 0.030 m/s, slope = 1.01, SD = 0.048 m/s). The data from ROI #11 have been circled

The quantitative comparison consists of a description of four representative ROIs displayed in Fig. 12 and a linear regression over all sixteen ROIs at time points over the entire cardiac cycle, see Fig. 13.

In Fig. 12a ROI A (ROI #5, slice 2) is close to the outlet of the right ventricle. The outlet is not visible in the CFD domain on the slice shown in Fig. 12a, but can be seen vaguely in the B-mode image behind ROI A. Both the CFD and VFI follow the same trend in velocity magnitude; a u-shaped curve with the lowest point just after 0.4 s. The magnitude for the CFD peaks at 0.18 m/s and has a minimum of 0.06 m/s. For the VFI the maximum is 0.24 m/s and the minimum is 0.11 m/s. In ROI A the CFD is consistently lower than the VFI and deviating slightly more than one standard deviation of the VFI.

ROI B (ROI #7, slice 3) is placed close to the moving boundary at the apex of the heart phantom where a large clockwise flow trend is observed in both CFD and VFI in Fig. 11a–d. Since this ROI is close to a moving boundary, the trend in velocity magnitude in ROI B is expected to be dominated by the changing proximity to the boundary. In the most extreme case, the moving boundary could cross the ROI position and report zero velocity during that period of time, however, this is not the case here. The velocity magnitude in the VFI is a reversed u-shaped curve with the peak around 0.3 s. The CFD is generally underestimating the VFI more in ROI B compared to ROI A, but it has a large local acceleration between 0.1 s and 0.2 s. The larger local acceleration in ROI B in the CFD could be explained by the larger spatial velocity gradient seen in the CFD data in Fig. 11a and b due to fluid being pushed into the ROI by the wall movement. The magnitude for the CFD predicted flow peaks at 0.33 m/s, and has a minimum of 0.10 m/s. For the flow obtained with VFI the maximum velocity is 0.31 m/s and the minimum velocity is 0.22 m/s.

ROI C (ROI #13, slice 5) is located close to the inlet jet, see Fig. 12e. The velocity magnitude trend is therefore expected to follow the trend of the volume flow rate, *Q*(*t*), applied to the system, which is shaped like a negative sine wave (see Fig. 6). There is a good correspondence between CFD and VFI in Fig. 12f since the velocity magnitude from the CFD simulation falls within one standard deviation of the VFI data. This is expected when data are extracted this close to the inlet because the inlet conditions for the flow simulation are based on the VFI measurement in close proximity to this location. The velocity magnitude for the CFD peaks at 0.38 m/s and has a minimum of 0.25 m/s. For the VFI the maximum is 0.40 m/s and the minimum is 0.26 m/s.

The last ROI, ROI D (ROI #15, slice 7), is placed in an almost cone-shaped part of the ventricle and close to the moving boundary, see Fig. 12g. The velocity magnitude trend is similar in both CFD and VFI. It starts and stops at 0.3 m/s over the flow cycle, where the largest deviation is a valley that occurs at $t = 0.35$ s in the CFD, and at $t = 0.42$ s in the VFI. The time shift of this valley can be explained by a small moving vortex that reaches and moves past ROI D in the CFD data set slightly before the same phenomena is observed in the VFI (See Online Resource 1 and 2, slice 7). The velocity magnitude of the CFD peaks at 0.32 m/s and has a minimum of 0.22 m/s. For the VFI the maximum is 0.34 m/s and the minimum is 0.17 m/s. The CFD velocity profile is within one standard deviation of the velocity profile obtained with VFI through the majority of the flow cycle, except in the interval from 0.4 s to 0.5 s.

#### Evaluation of the Out-of-Plane Component

The CFD data provides velocity information in all three spatial dimensions, so to complete the evaluation of the full velocity field, the quantitative data for all three velocity components in the ROIs presented in Fig. 12 are presented here. Table 2 lists the average values for each velocity vector component in each of the four ROIs. The evolution in time of the three velocity components in each ROI is shown in Online Resource 3.

**Table 2:** Average velocity magnitudes for all three velocity vector components within ROIs

	$\langle u \rangle$ [m/s]	$\langle v \rangle$ [m/s]	$\langle w \rangle$ [m/s]
ROI A	0.097	0.064	− 0.043
ROI B	0.119	− 0.095	− 0.159
ROI C	− 0.327	0.026	0.019
ROI D	− 0.196	− 0.084	− 0.192

Here $\langle u \rangle$, $\langle v \rangle$ and $\langle w \rangle$ are the average over one full flow cycle

In ROI C, which is closest to the inlet, the out-of-plane component $\langle v \rangle$ has a magnitude corresponding to 7.95% of the dominating in-plane velocity component $\langle u \rangle$. For ROIs A and B the out-of-plane component is 66.0% and 59.8% of the dominating in-plane velocity component respectively. Lastly, in ROI D which is closest to the apex of the heart phantom, $\langle v \rangle$ is 43.9% of the dominating in-plane velocity component.

### Global Evaluation

To complete the comparison and evaluate the performance of the proposed pipeline, a global evaluation is made based on all 16 ROIs. The time dependent spatial mean velocity magnitude within each ROI is divided into 11 non-overlapping time bins. The temporal means of the resulting 176 time bins are compared between CFD and VFI in a scatter plot with a fitted linear regression line. The scatters in Fig. 13 are the 176 CFD time bins plotted against the 176 VFI time bins.

A group of 11 blue scatters in Fig. 13, all belonging to ROI #11, are marked with a circle to indicate that they are considered as outliers. The large deviations in ROI #11 are thought to be an effect of the inlet corrections described in “Computational Fluid Dynamics” section since this ROI is positioned in the transition between the inlet pipe which has a rigid wall and the RV volume that has a moving wall boundary. An evaluation including all ROIs is made as well as an evaluation excluding ROI #11. The evaluation consists of a linear regression fitted to the scatters in Fig. 13 and a calculation of the standard deviation. The resulting regression numbers are presented in Table 3. From this, as expected, excluding the suspected outliers from ROI #11 the $R^2$ improves from 0.809 to 0.823. This is also reflected in the slope of the regression line, which improves from 1.09 to 1.01, as well as in the SD on the velocity magnitude. Here the SD is reduced from 0.06 to 0.048 m/s. In both regressions the slope is close to 1 with intercept below zero at − 0.039 and − 0.030 m/s respectively. This indicates that the output of the CFD pipeline consistently underestimates the velocity magnitude compared to VFI, and that the level of underestimation is independent of the measured velocity magnitude.

**Table 3:** Regression numbers

	$R^2$	Intercept [m/s]	Slope	SD [m/s]
All ROI’s	0.809	− 0.039	1.09	0.060
Excluding ROI #11	0.823	− 0.030	1.01	0.048

## Discussion

The results presented in this paper show a CFD pipeline applied for the first time on a dynamic heart phantom and compared to a highly controlled ultrasound VFI measurement of the same phantom under the same conditions. This work flow facilitates validation of cardiac flow properties based on directly obtained flow-based metrics. The CFD pipeline is based on the movement of the phantom ventricle, which is gathered from CTA and volumetric image registration. The inlet was a prescribed time dependent velocity calculated to provide the same volume flow rate as in the VFI measurements. A qualitative comparison, based on in-plane velocity direction and magnitude, showed overall correspondence when looking at the locations close to the inlet and outlet. When comparing the results in positions further away from the inlet towards the apex the similarity was less. The same tendency was observed for the quantitative results. A quantitative comparison, based on 11 non-overlapping time bins in all 16 ROIs showed the CFD estimates to be consistent, however with a negative bias and a SD of 10% at the highest measured values. Although the standard deviation of the CFD estimates seems large, the comparison is based on a pipeline where the registration approach has not yet been optimised. An important optimisation to the registration is to consider the temporal dimension in the registration instead of treating each registration as independent. Looking at the quantitative results in Fig. 12, the hypothesis of this paper has not be met for all 16 ROIs.

The bulk blood flow fluid dynamics inside the RV presented here is solved using geometry-prescribed CFD, where the numerical technique is based on the ALE formulation. Others have modelled the fluid dynamics in the heart using single particle hydrodynamics (SPH) [2, 25]. The SPH is a mesh-free finite element method, which handles the solution of the fluid and solid mechanics in one algorithm as it is a purely Lagrangian formulation. This is advantageous when valves are part of the model geometry [18]. The numerical computation here is a two step procedure where the mesh movement is handled according to a Lagrangian description, and the CFD is handled in an ALE formulation of the Navier–Stokes equations, which is a mesh-based method. This is done to lower the computational complexity and reduce the computation time. The use of the ALE method is not straight forward in the case where heart valves are included in the model geometry due to the intricate fluid–structure interaction governed by the rapid movement and slender structure of the valves.

The overall design of the pipeline has been developed with application to in vivo data in mind, and the first step is to perform validation in a phantom to provide a higher quality comparison compared to in vivo. The presented phantom setup is therefore a crucial tool for developing the CFD pipeline further and bring it to a stage where it can be applied to in vivo data. With more optimisation the current discrepancies between measurement and simulation can be reduced.

The phantom is measured with ultrasound for validation, because there are no anatomical restrictions, and the ultrasound transducer can be mounted in a fixture, adding spatial information and consistency to the measurements. The phantom measurements are in parallel planes with 5 mm between them, which would be very hard to obtain in vivo, because the transducer must be placed in contact with the skin, and either between or under the ribs.

Since the CFD pipeline estimates three dimensional flow velocities, the presented validation only compares two of the three dimensions. Neglecting the out-of-plane component in the analysis can be valid in flow of low complexity. However, the flow in the heart has a high complexity. In this study, the out-of-plane component at the inlet was 7.95% of the dominating in-plane component magnitude. This is because the fluid has not yet entered the RV and thus flow in a laminar configuration with a slightly blunt parabolic profile. But the further the fluid advances inside the RV away from the inlet, the out-of-plane component becomes more dominant, especially during the stretching from fully compressed to the initial position of the phantom (see Online Resource 3). The fixture shown in Fig. 2 is capable of measuring planes orthogonal to the presented data set, and can thereby obtain velocity data for the out-of-plane velocity component. But this is not a trivial measurement to perform. For the ultrasound probe used here, the sample volume for a velocity estimate is at best $1 \times 3 \times 0.5$ mm^3^ and up to 10 mm in the *y*-direction due to the probe’s acoustic lens. While it would be technically possible to measure the out-of-plane component by rotating the probe 90 degrees and thereby form a full 3D velocity vector, this anisotropic sample volume would make the combination of the *x* and *z* components from one measurement with the *y* component from a second measurement highly uncertain. An alternative could be to acquire the ultrasound data in 3D to get the full velocity field. Currently, there are techniques to do 3D ultrasound flow estimation [3, 10], with only a few examples of imaging and quantification of complex flow [30]. These techniques still lacks detailed performance evaluation before they are applicable as ground truth for validation of flow simulation pipelines like the one presented here.

The setup here even allows changing parameters, such as heart rate, stroke volume, beat-to-beat variations, etc., to create best-case and worst-case scenarios for targeted troubleshooting during development of the pipeline before moving to in vivo validation. Most importantly, the prescribed phantom movement and flow patterns are repeatable. This allows for repeated measurements with minimal inter-measurement variation, which is why averaging over 210 cardiac cycles is acceptable in the phantom measurements, even though it is listed as a disadvantage for in vivo methods.

The purpose of this work is to present a novel CFD pipeline and validation setup for development of cardiac flow simulation models. The metrics for evaluating accuracy in this paper are therefore designed to compare the CFD flow patterns to the VFI measurements as transparently as possible. Other publications have largely been comparing metrics already used in the clinic such as stroke volume, peak flow rate, kinetic energy at peak systole, etc. [16]. While these metrics are directly translatable and valuable in clinic, they are either directly derivable or closely related to geometry changes instead of the flow patterns themselves. These metrics are often an integration over a large volume or a wide time span. The work presented in this paper is a smaller, more scrutinised, step in the intra-cardiac CFD pipeline development, focusing directly on the flow patterns. Metrics derived from CFD simulation models can only truly be trusted, if it is certain that they are derived directly from the actual blood flow patterns. When this is achieved, CFD simulations can be a valuable addition to existing clinical methods, adding new important flow based metrics to the diagnosis of cardiac diseases, as well as providing a more detailed post-operative evaluation as part of the treatment follow-up.

### Limitations

An important limitation to all phantom validation is that it only reflects the performance of the method when applied to the phantom. The inlet flow was applied using a simple commercially available impeller pump allowing for application of constant flow only and less exact control of the delivered flow rate. The events of systole and diastole were governed by the cyclic motion of the phantom applied through the actuator rod, where the compression represents the systolic phase, and the diastolic phase is when the the phantom returns to its starting position. In combination with the fact that the phantom is designed without valves, this resulted in that the pump was significantly affected by the changes in after-load caused by the compression of the phantom. The inlet conditions were therefore extracted from the ultrasound VFI measurements to obtain the correct volume flow rate. The applied volume flow rate is low compared to the physiological flow rate [7]. The nominal flow rate of the pump is 5 L/min, corresponding to the average cardiac output in an adult. But this flow rate was significantly reduced because the afterload on the pump outmatched what the pump was designed for. Misalignment of the imaging plane used for estimation of the spatial mean velocity caused an underestimation of the true velocity equivalent to 80% of the actual spatial mean velocity, which corresponds to that the imaging plane is at 89.4% of the true vessel diameter. This, however, will only have a little impact on the estimated volume flow rate. It has been shown that a 10% off-axis error for the imaging plane results in approximately a 5% underestimation of the volume flow rate [13]. This causes the inlet conditions to be a function of the VFI measurement as well as the segmentation of the inlet geometry, which reduces the control of the phantom. Thus, the pipeline is not validated against a completely independent measurement. Future validation measurements should use a calibrated gear-head pump, which is less sensitive to changes in after-load and can control the volume flow rate to match in vivo cases. This will simplify the inlet boundary condition in the CFD model, and increase the control of the heart phantom.

The standard deviation of the velocity magnitude, excluding ROI #11, is 0.048 m/s. Relative to the highest measured velocity this SD is around 10% which is excellent for such complicated flow as investigated here. Additionally, considering that the wall movement in the presented CFD pipeline is based on an non-optimised registration approach, the flow simulation pipeline has the potential to obtain an even higher precision through optimisation of the registration approach.

Evaluating quantitative results like in Fig. 13 and Table 3 from a controlled phantom setup is going to be an essential validation tool during future pipeline development. The general underestimation in velocity magnitude, represented by the intercept and slope of the regression, could be a consequence of the inlet estimation. The registration approach in this work was performed in the simplest way possible that still provided acceptable results. Considering the presented method is founded on a geometry prescribed CFD simulation of the flow behaviour, the wall movement is the most important input to the model for accurate results. The presented approach performs 19 independent registrations, between first volume and all other volumes, however the registrations are not truly independent, and the temporal information should be included in the registration [22].

## Conclusion

A complete pipeline for geometry prescribed CFD simulation of intra-cardiac blood flow patterns has been applied on a dynamic heart phantom for validation purposes. The qualitative comparison with ultrasound VFI data shows overall similarities in flow direction and location of vortexes. The quantitative data showed similarity in time progression of velocity magnitude in 16 different ROIs with a general tendency of underestimation: $R^2=0.823,$ intercept = − 0.030 m/s, slope = 1.01, and SD = 0.048 m/s. This direct comparison of flow properties and patterns shows that the flow simulation pipeline applied here produce accurate flow patterns in regions close the inlet and outlet, since the velocity magnitude is within one SD of the reference VFI data. But it also shows that in regions far away from the well-controlled boundary conditions the pipeline fails to produce accurate results. By optimising the registration method and apply well-controlled volume flow in the experimental setup, the output of the pipeline can become more reliable and applied to in vivo cases.

## Supplementary Information

Below is the link to the electronic supplementary material.

Supplementary file 1 (pdf 2574 KB)

Supplementary file 1 (pdf 1334 KB)

Supplementary file 1 (pdf 161 KB)
