# Supplementary material for: Intra-Cardiac Flow from Geometry Prescribed Computational Fluid Dynamics: Comparison with Ultrasound Vector Flow Imaging
Source: Cardiovasc Eng Technol. 2023 Jun 15;14(4):489–504. doi: 10.1007/s13239-023-00666-2 (PMC10465406; doi:10.1007/s13239-023-00666-2)

# Intra-Cardiac Flow from Geometry Prescribed Computational Fluid Dynamics: Comparison with Ultrasound Vector Flow Imaging

Journal: Cardiovascular Engineering and Technology

Authors: Rasmus Hvid[1], Matthias Bo Stuart[1], Jørgen Arendt Jensen[1] and Marie Sand Traberg[1*]

[1] Department of Health Technology, Technical University of Denmark, 2800 Kongens Lyngby, Denmark

*Corresponding author: Marie Sand Traberg, msene@dtu.dk

## CFD in-plane velocity, slice 1

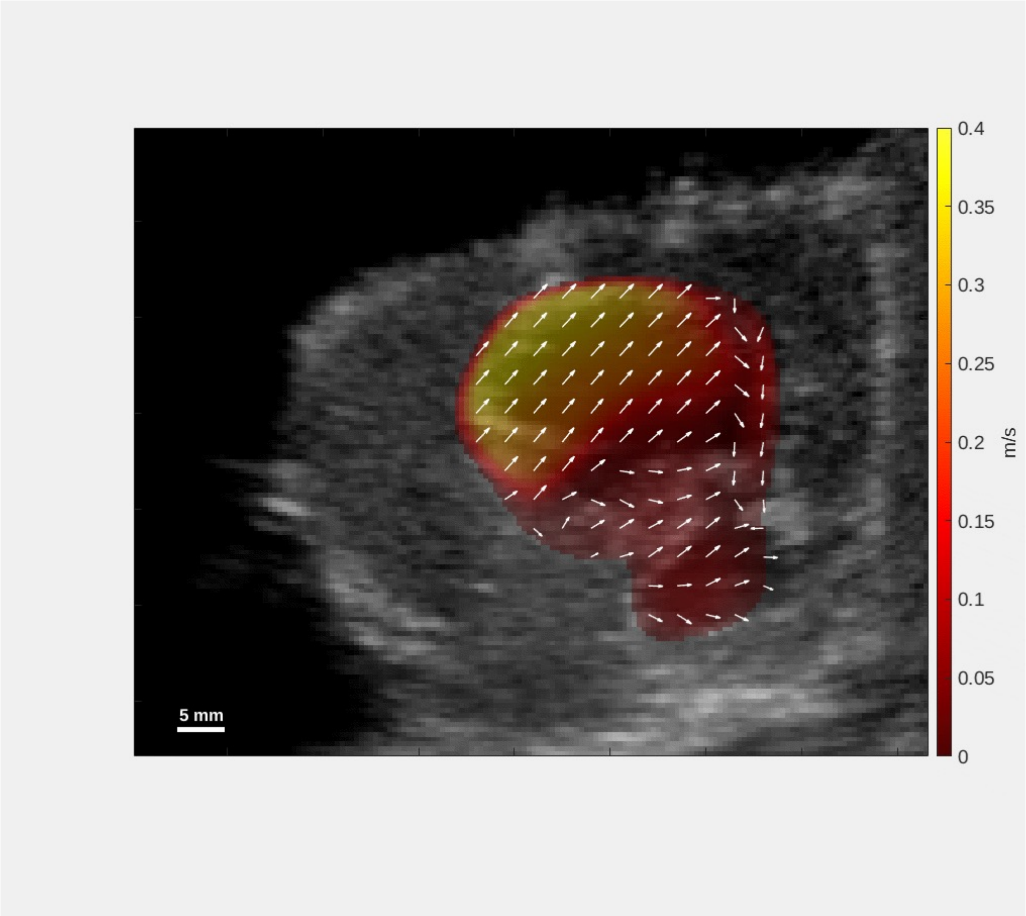


## VFI in-plane velocity, slice 1

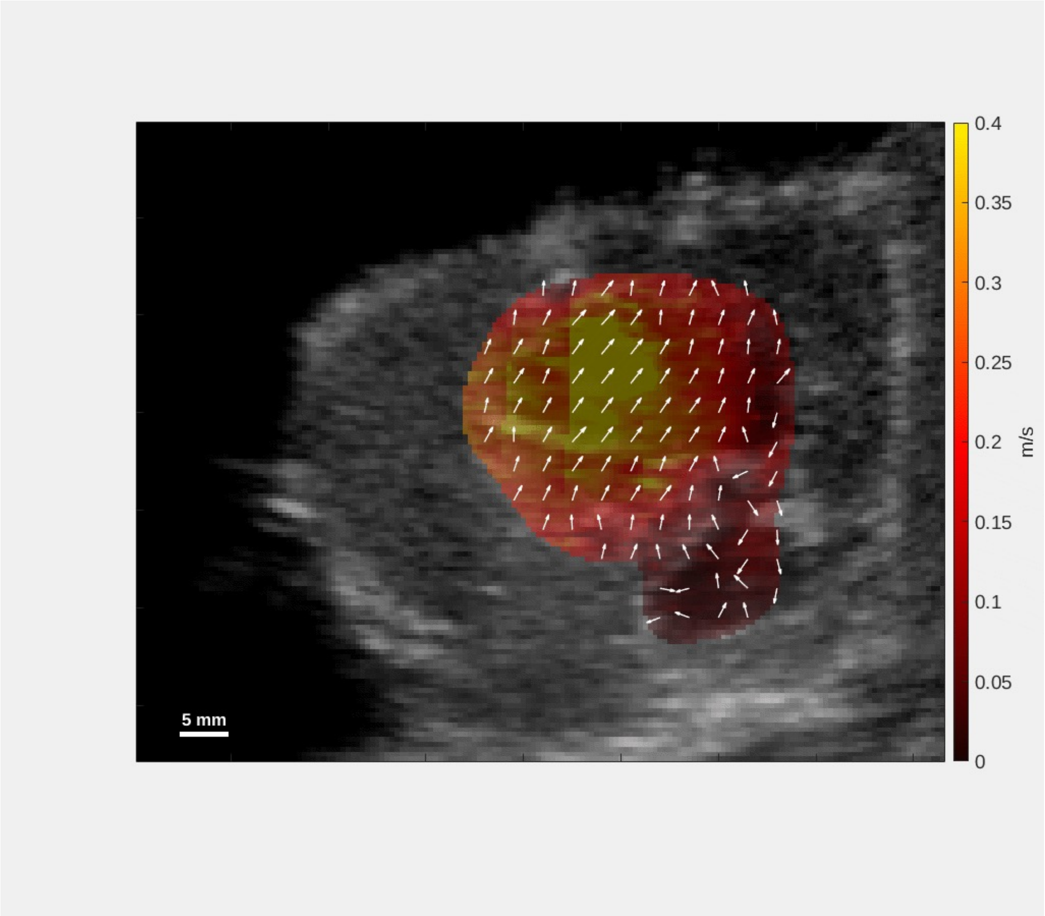

**CFD in-plane velocity, slice 2**

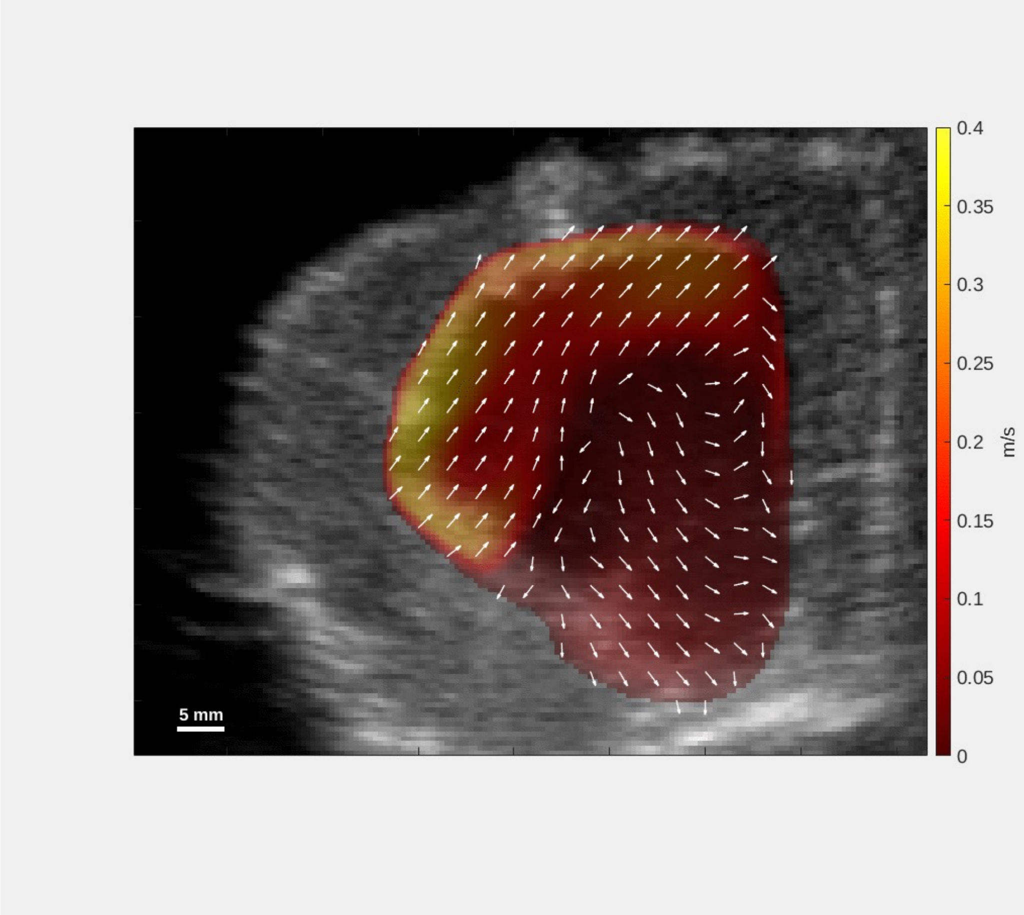


**VFI in-plane velocity, slice 2**

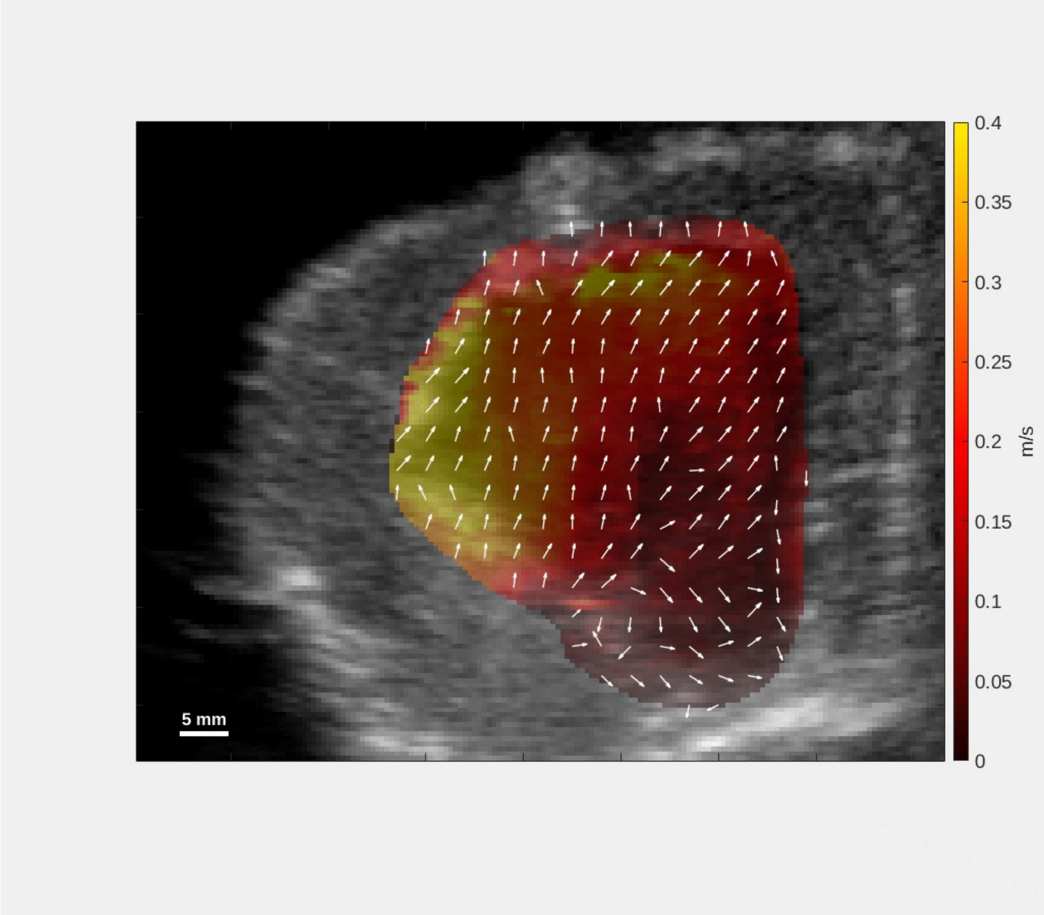

## CFD in-plane velocity, slice 3

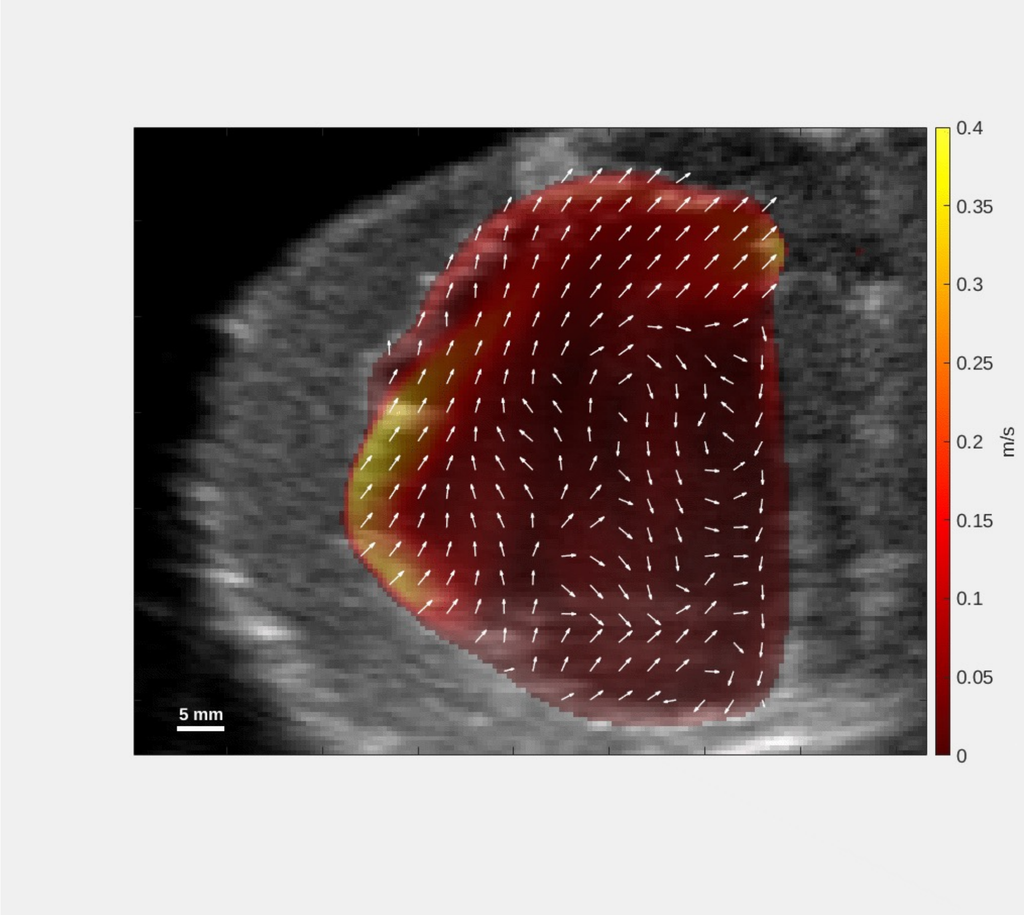


## VFI in-plane velocity, slice 3

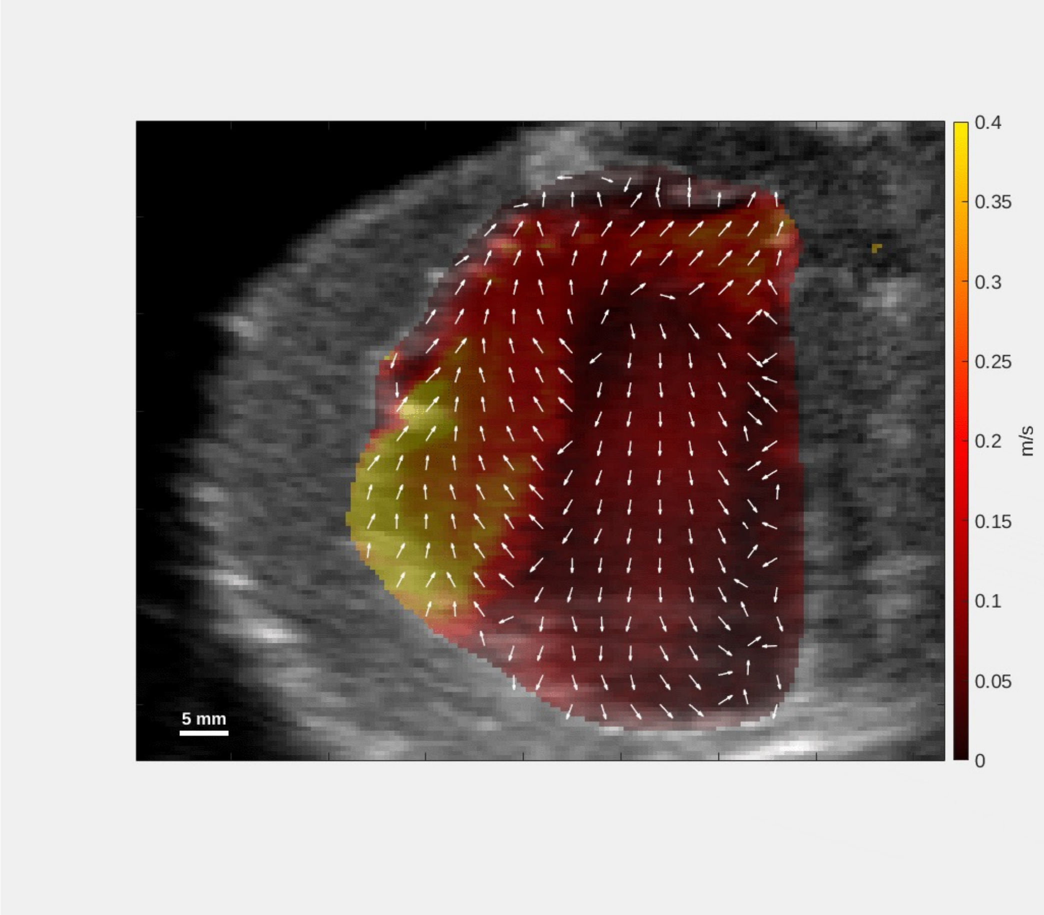

## CFD in-plane velocity, slice 4

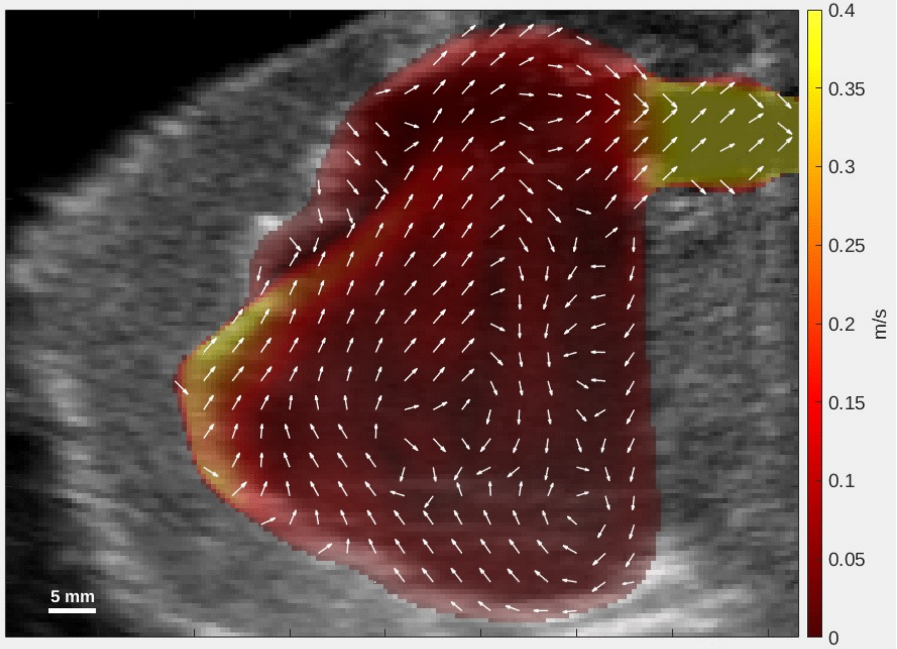


## VFI in-plane velocity, slice 4

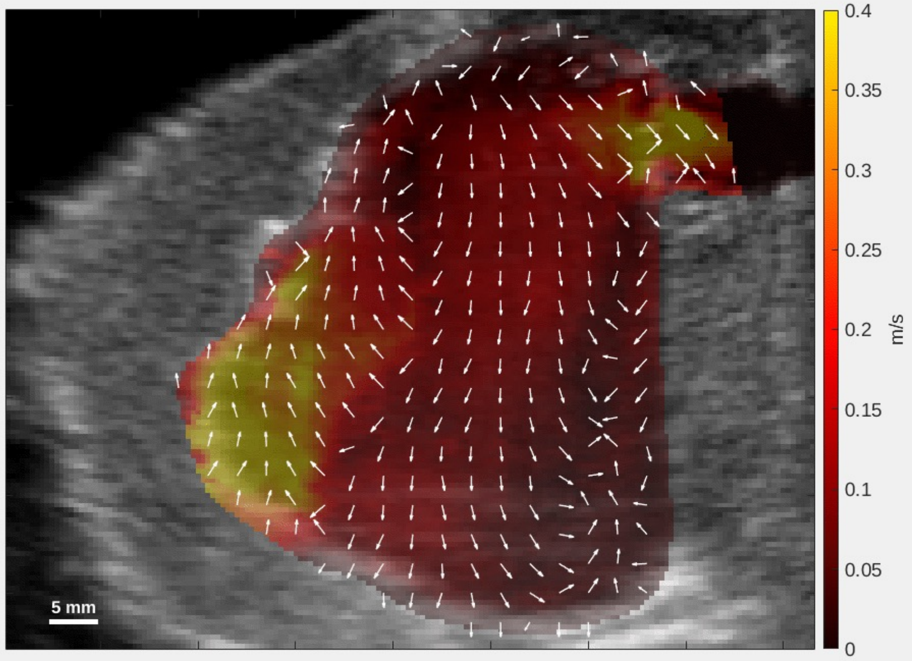

## CFD in-plane velocity, slice 5

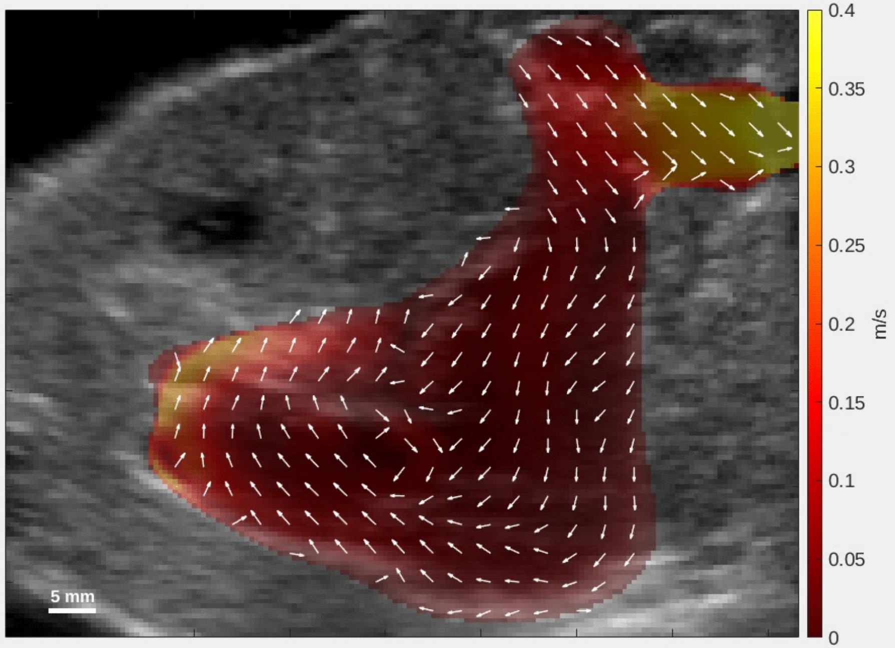


## VFI in-plane velocity, slice 5

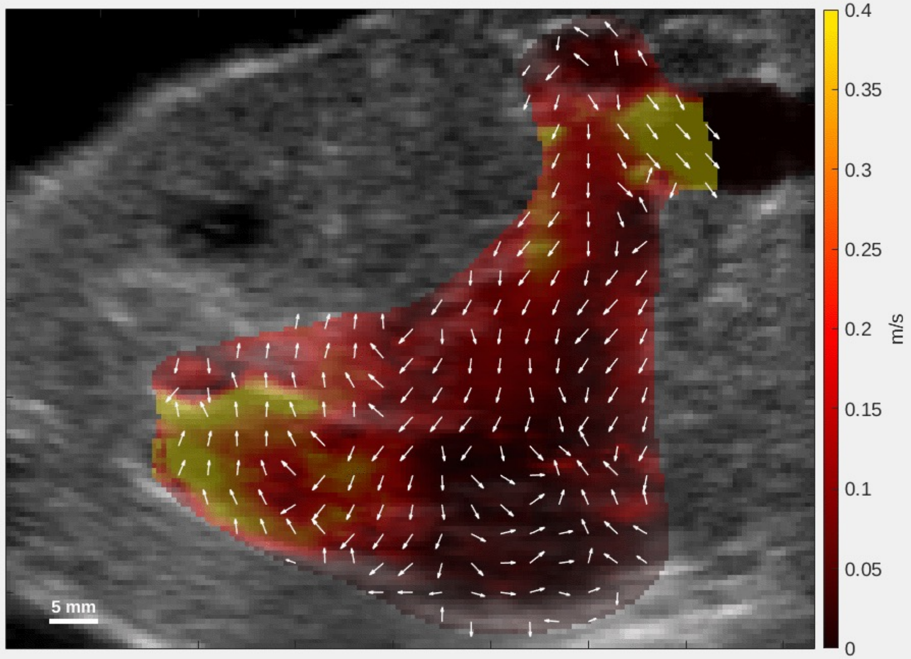

## CFD in-plane velocity, slice 6

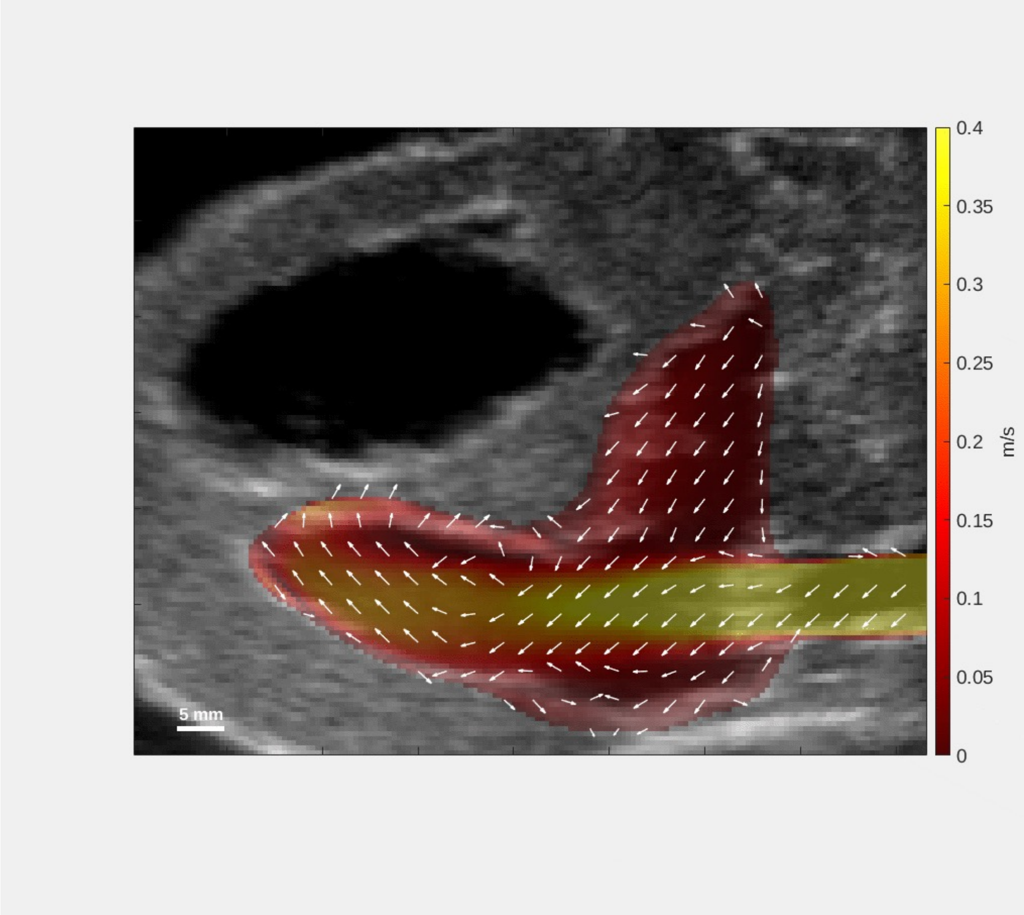


## VFI in-plane velocity, slice 6

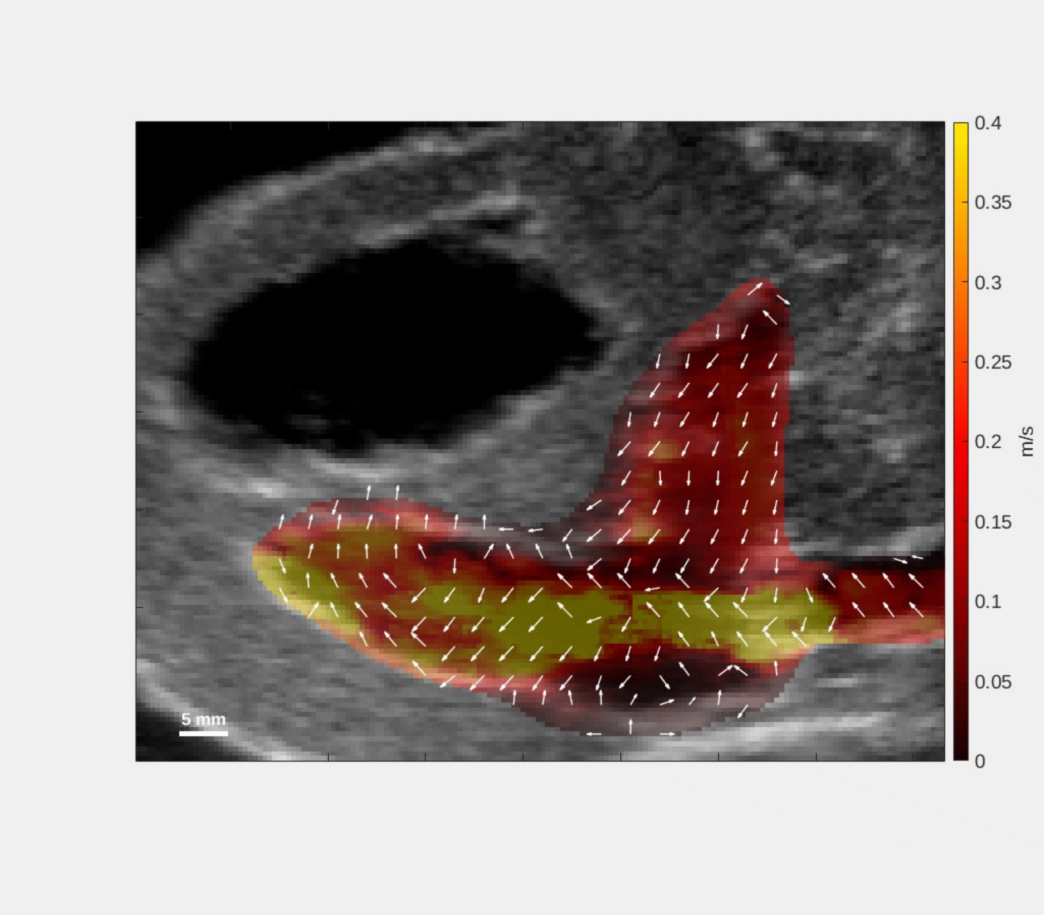

## CFD in-plane velocity, slice 7

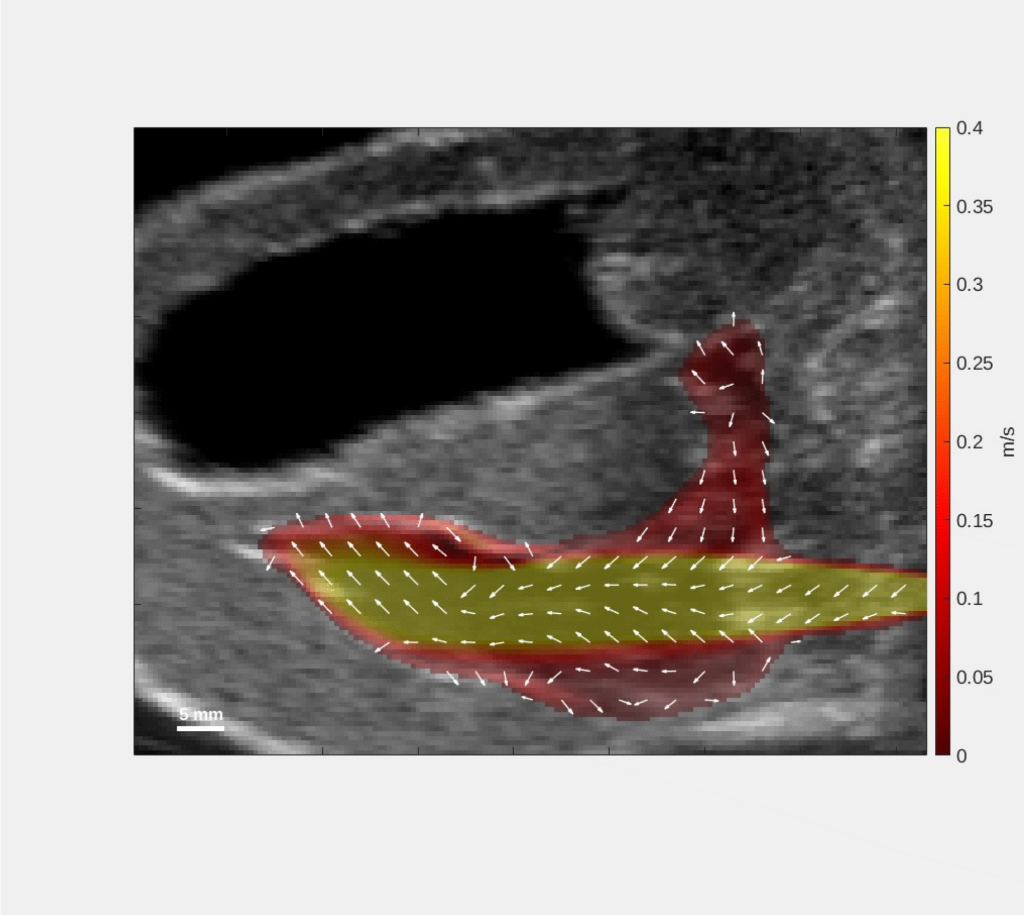


## VFI in-plane velocity, slice 7

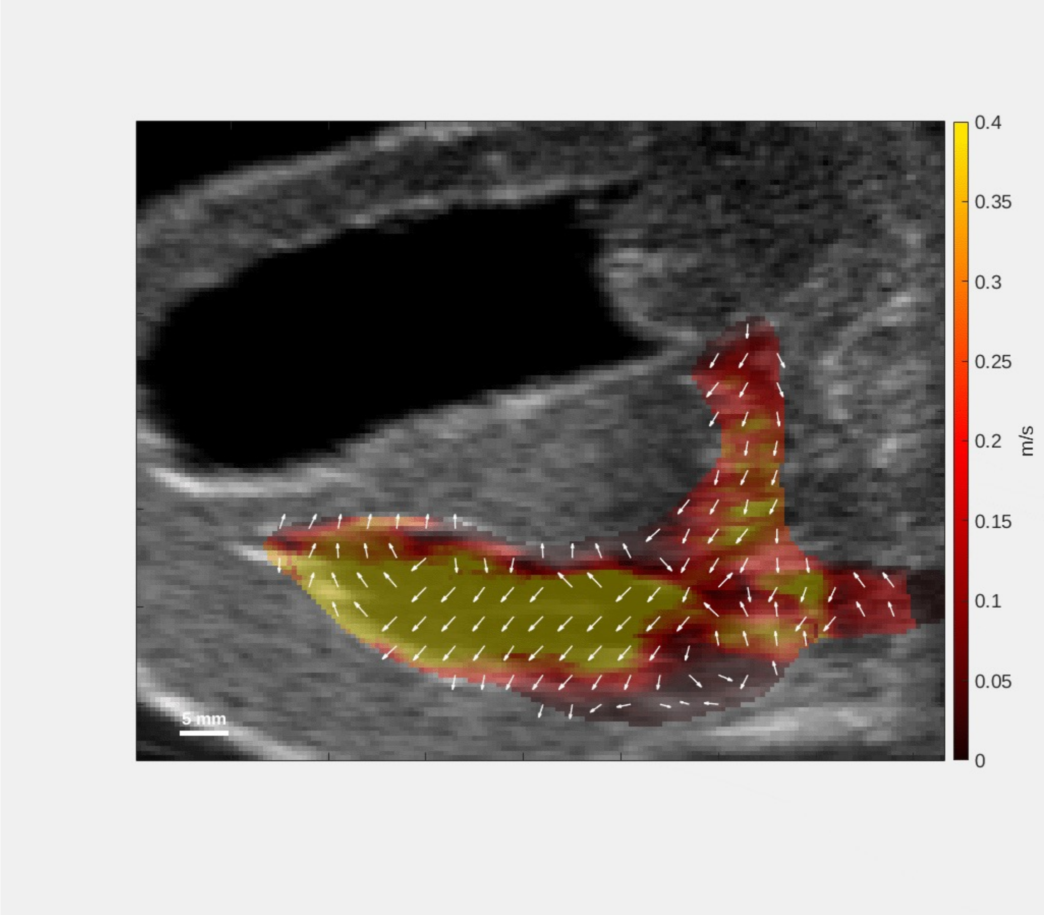

## CFD in-plane velocity, slice 8

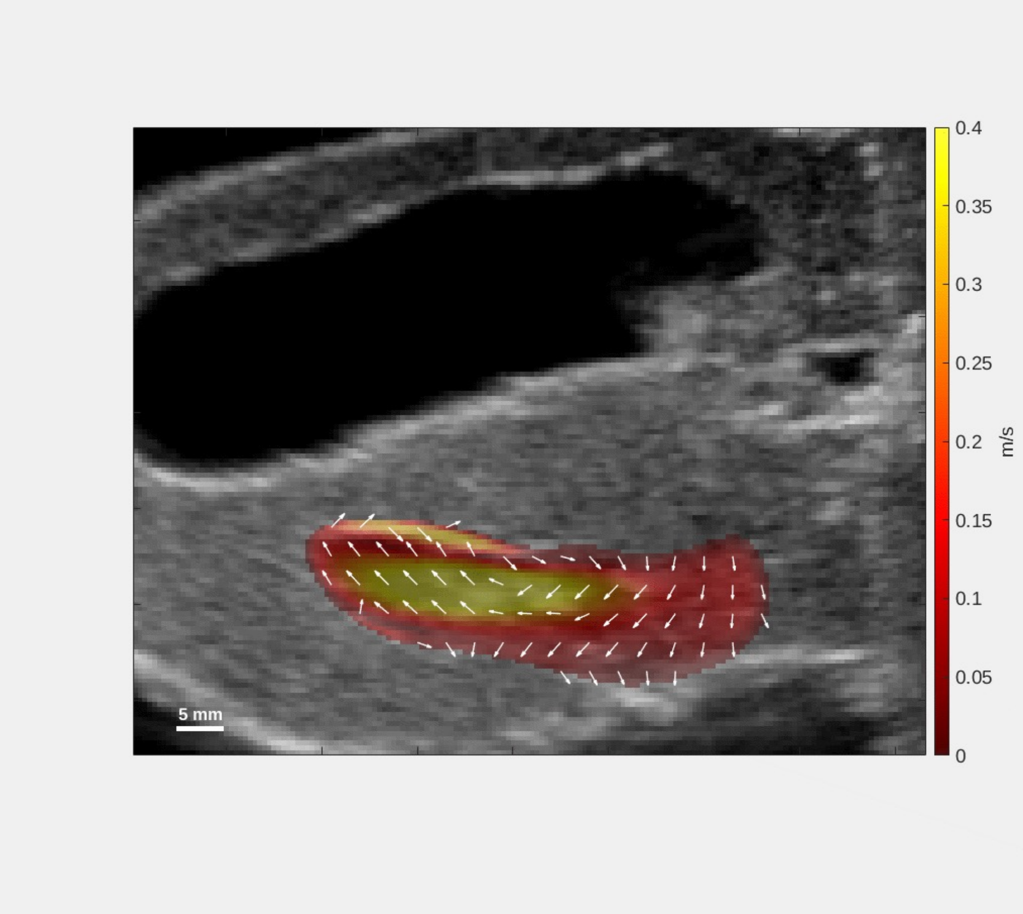


## VFI in-plane velocity, slice 8

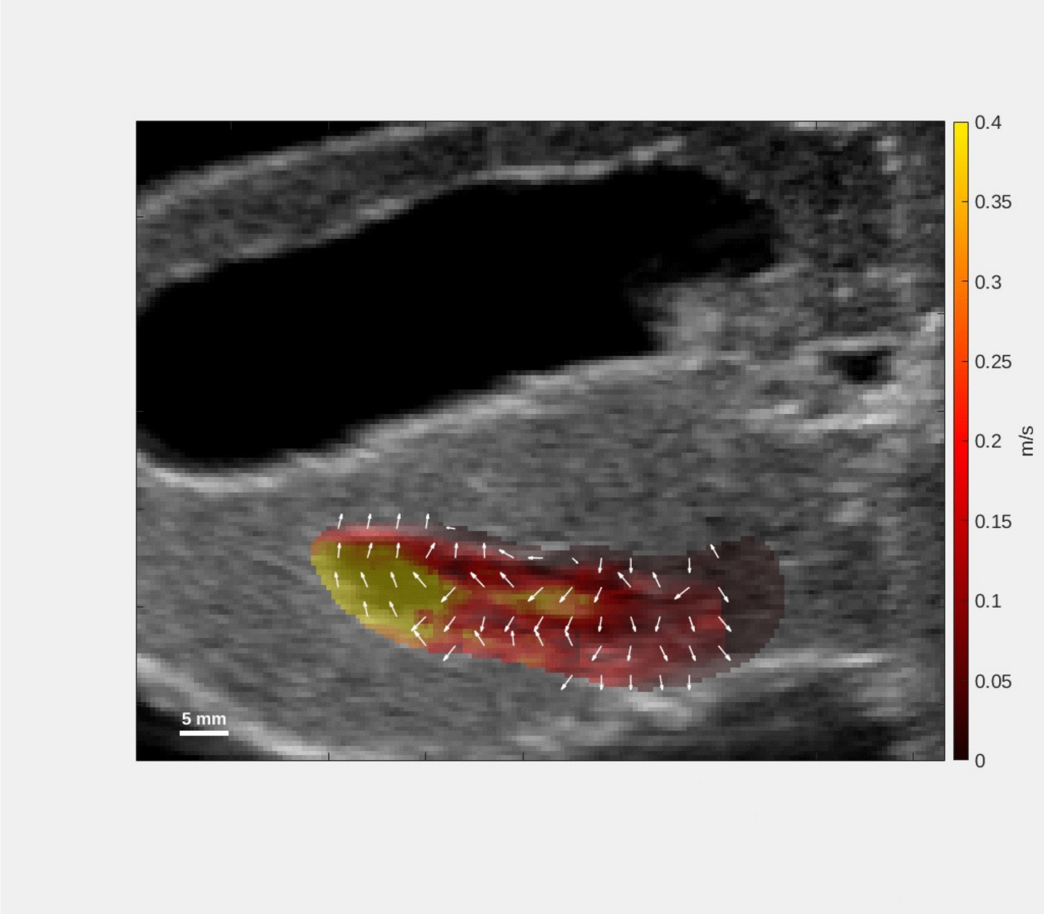

Supplement: Supplementary file 1 — (pdf 2574 KB) [file 13239_2023_666_MOESM1_ESM.pdf]
