# Supplementary material for: Intra-Cardiac Flow from Geometry Prescribed Computational Fluid Dynamics: Comparison with Ultrasound Vector Flow Imaging
Source: Cardiovasc Eng Technol. 2023 Jun 15;14(4):489–504. doi: 10.1007/s13239-023-00666-2 (PMC10465406; doi:10.1007/s13239-023-00666-2)

Intra-Cardiac Flow from Geometry Prescribed Computational Fluid Dynamics: Comparison with Ultrasound Vector Flow Imaging

Journal: Cardiovascular Engineering and Technology

Authors: Rasmus Hvid[1], Matthias Bo Stuart[1], Jørgen Arendt Jensen[1] and Marie Sand Traberg[1*]

[1] Department of Health Technology, Technical University of Denmark, 2800 Kongens Lyngby, Denmark

*Corresponding author: Marie Sand Traberg, msene@dtu.dk

ROI #1

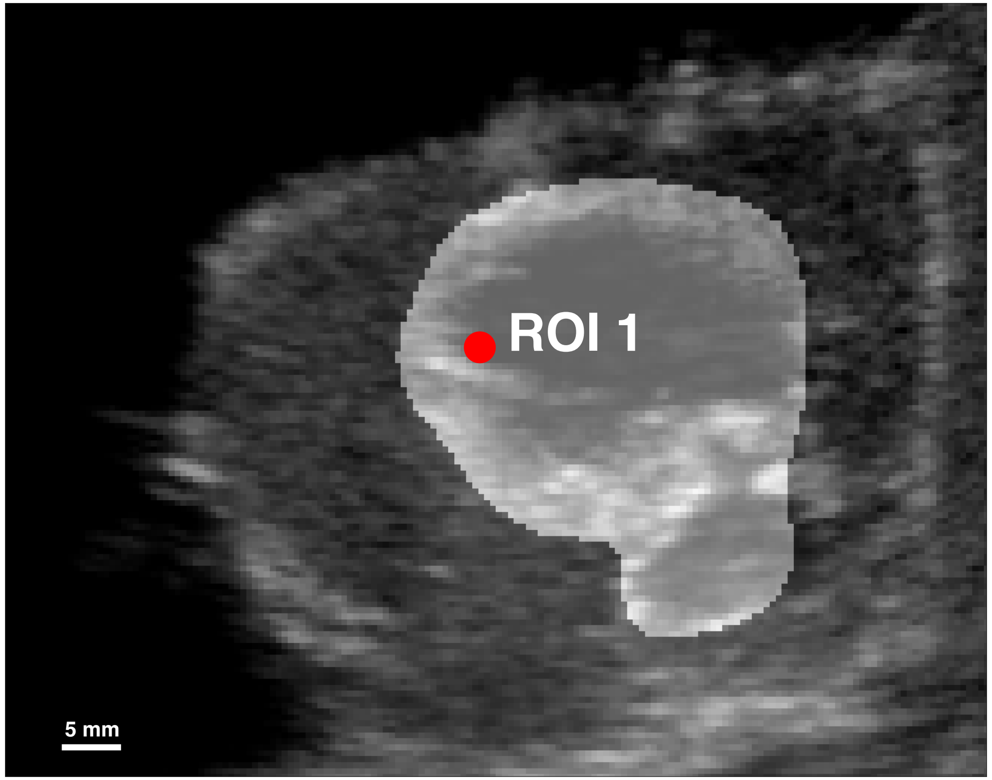

ROI #2

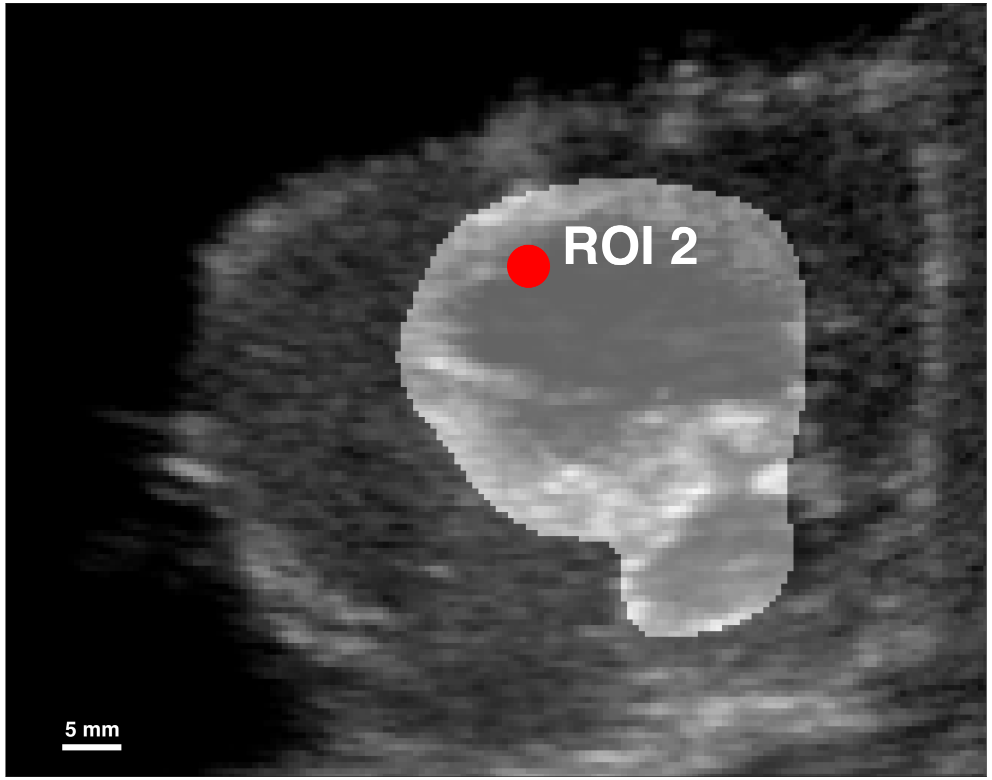

ROI #3

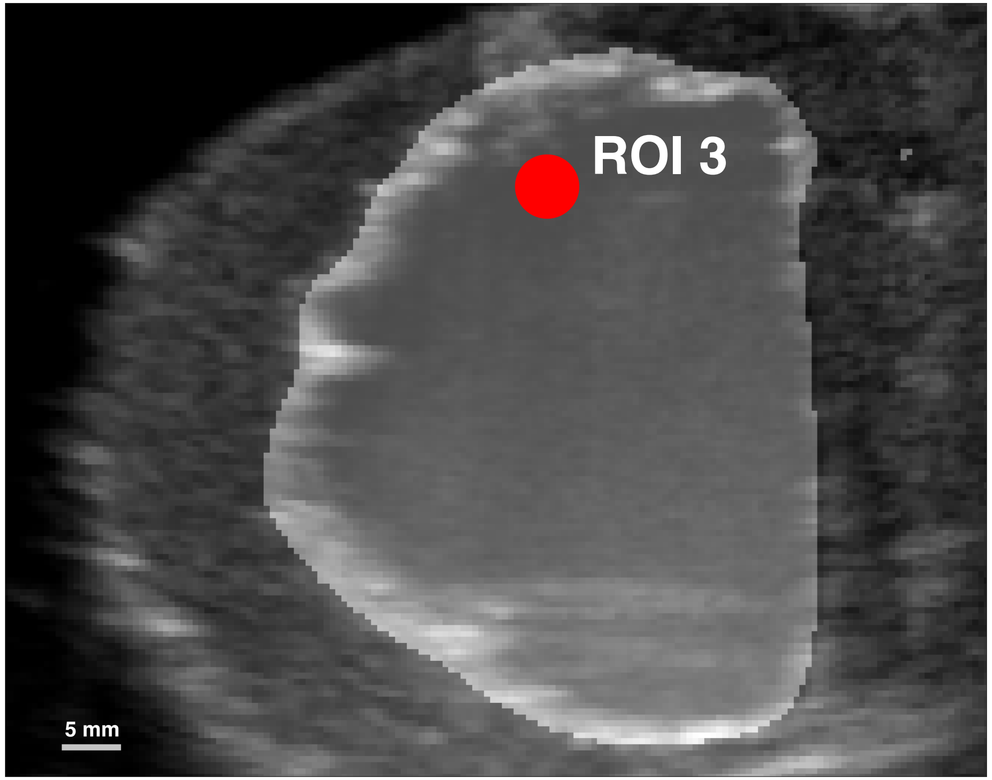

ROI #4

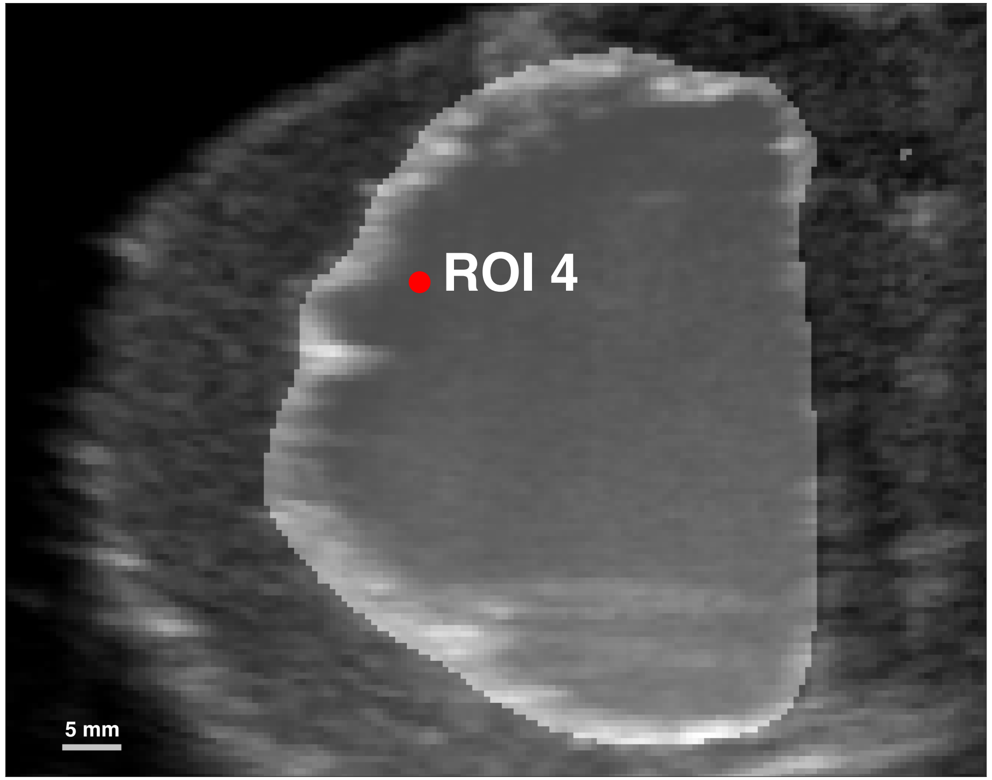

ROI #5

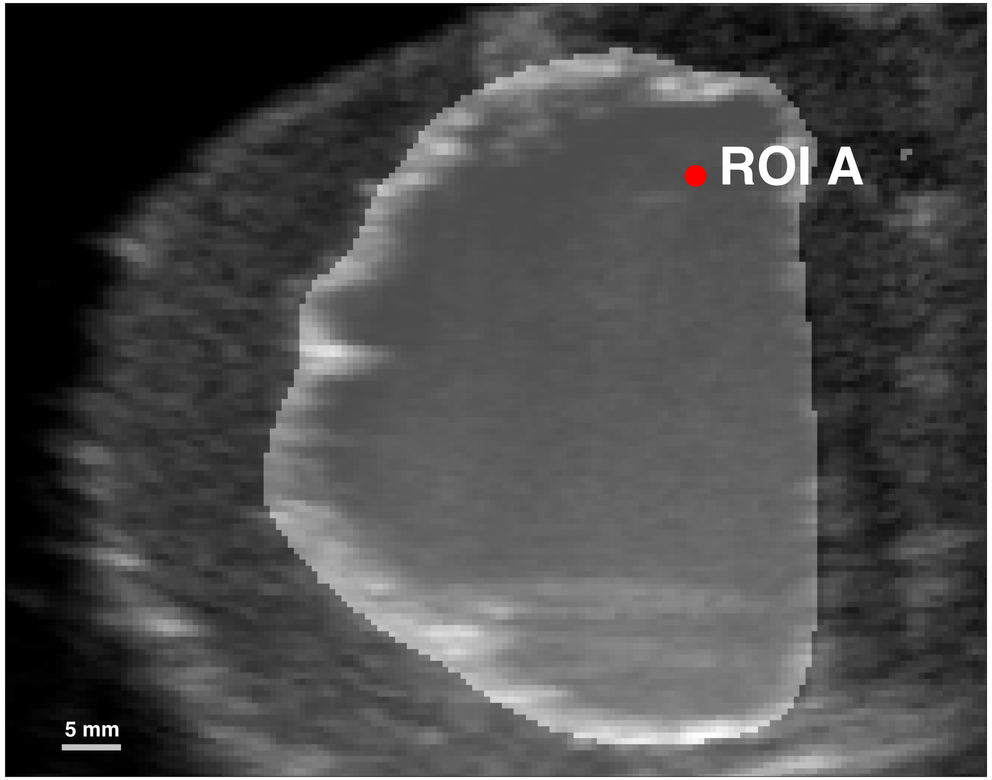

ROI #6

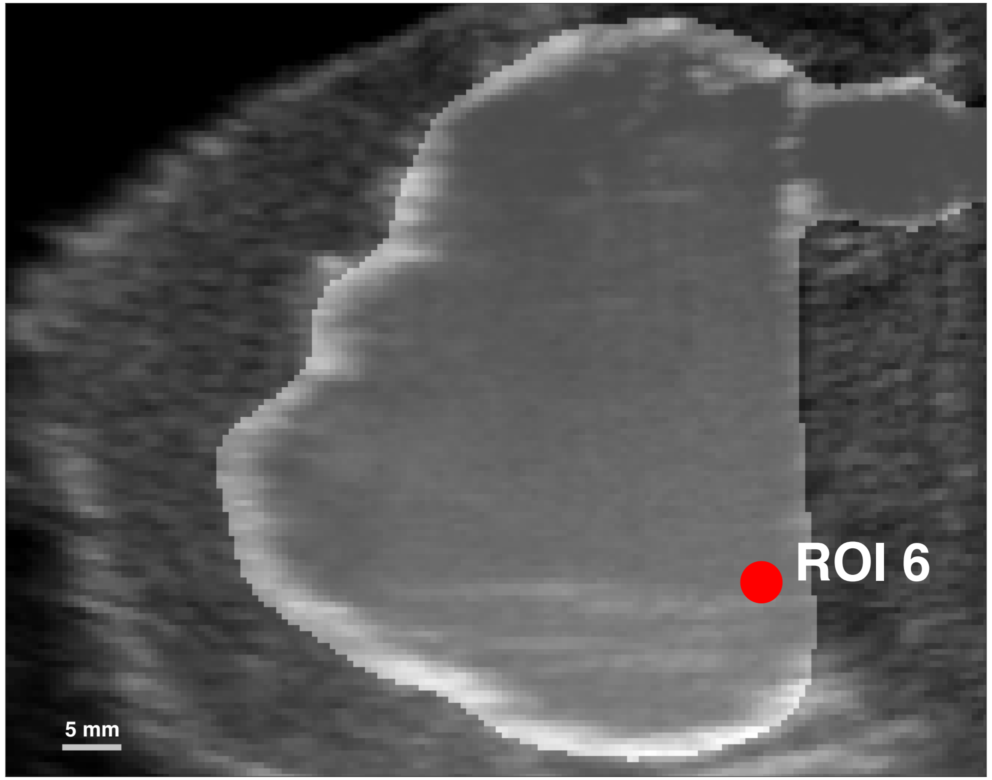

ROI #7

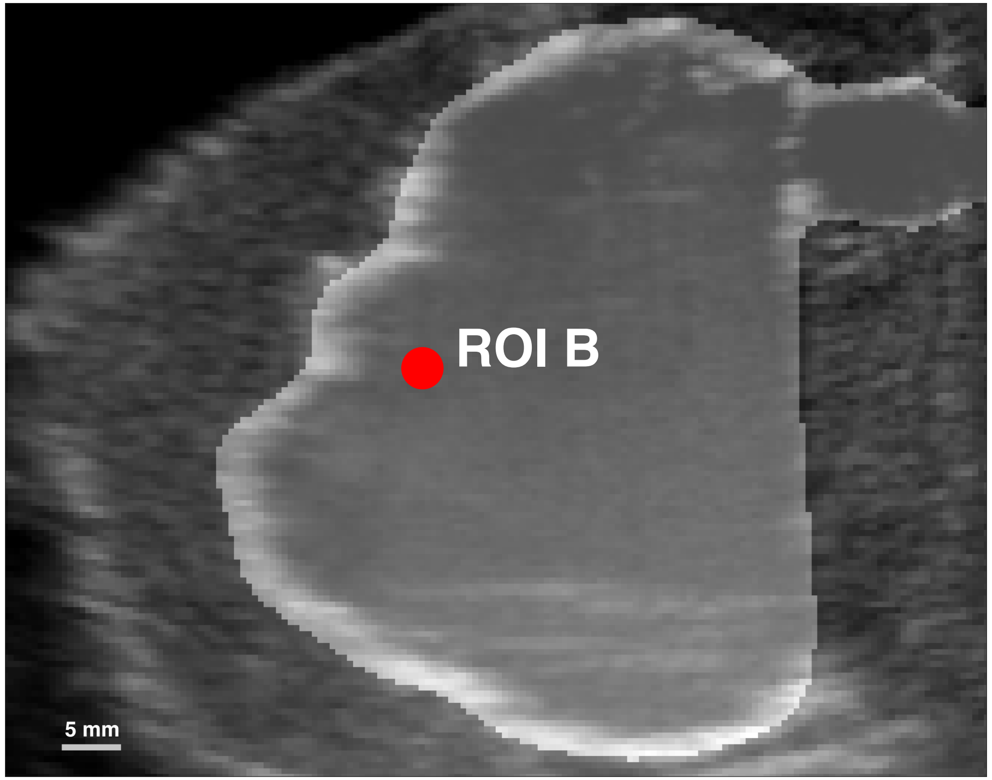

ROI #8

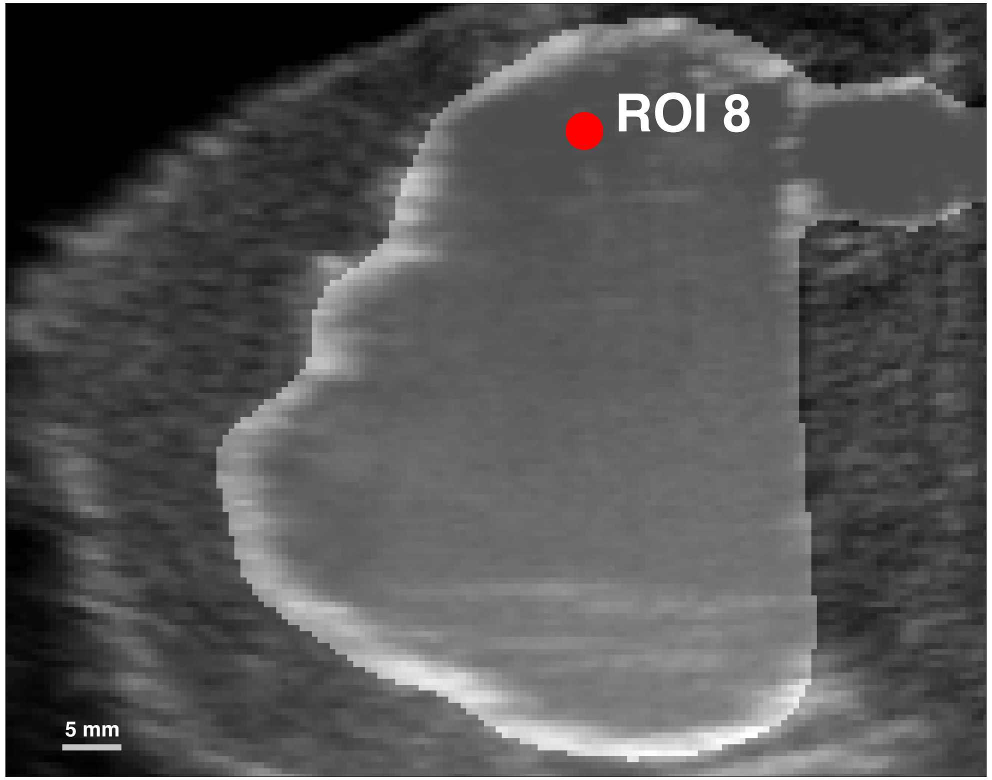

ROI #9

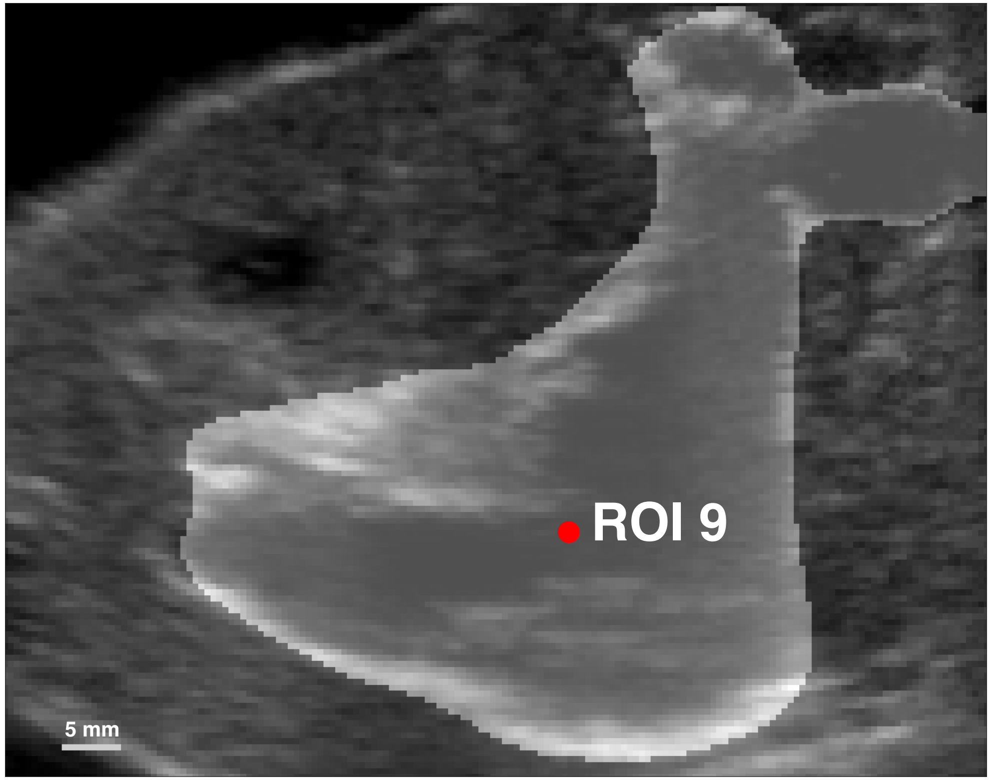

ROI #10

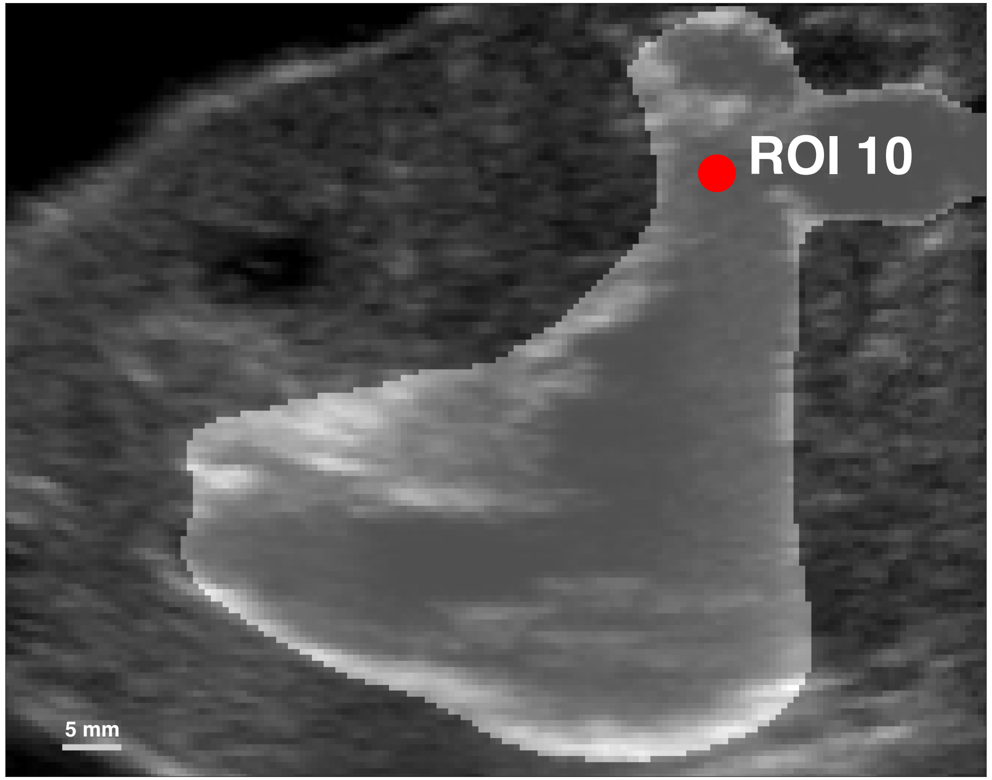

ROI #11

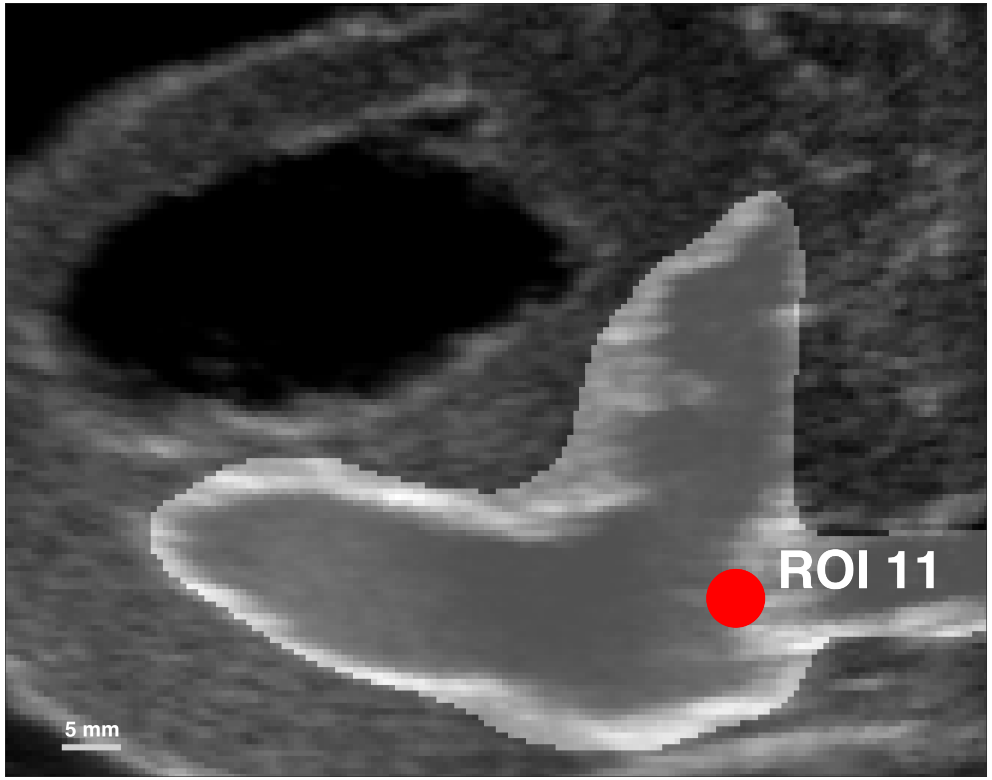

ROI #12

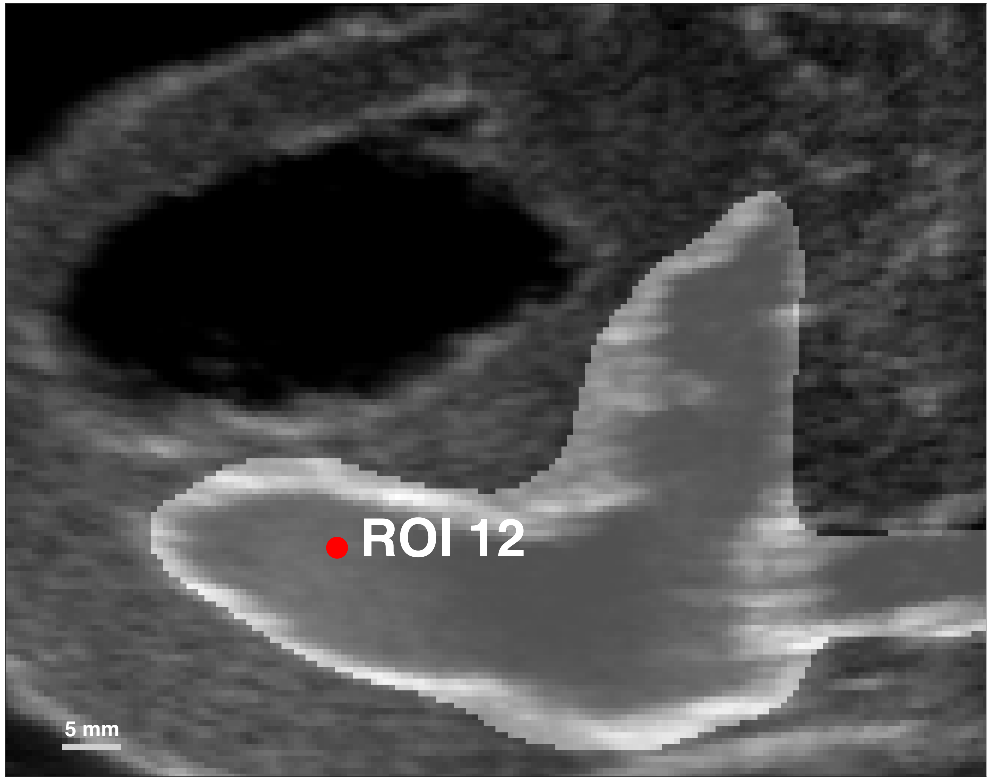

ROI #13

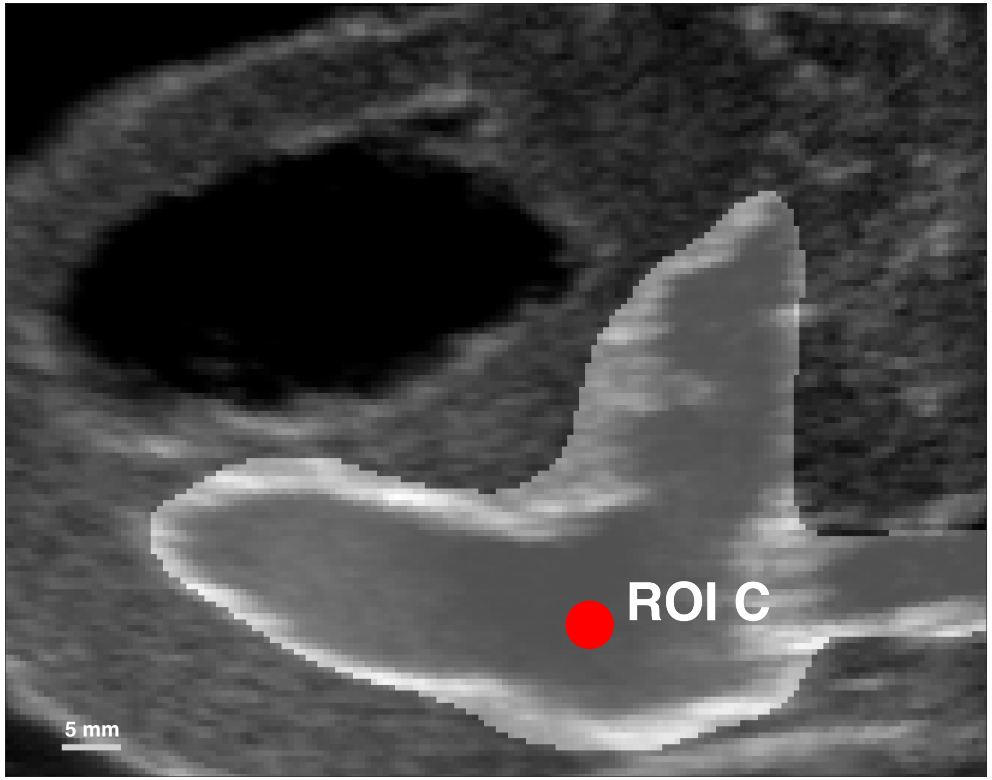

ROI #14

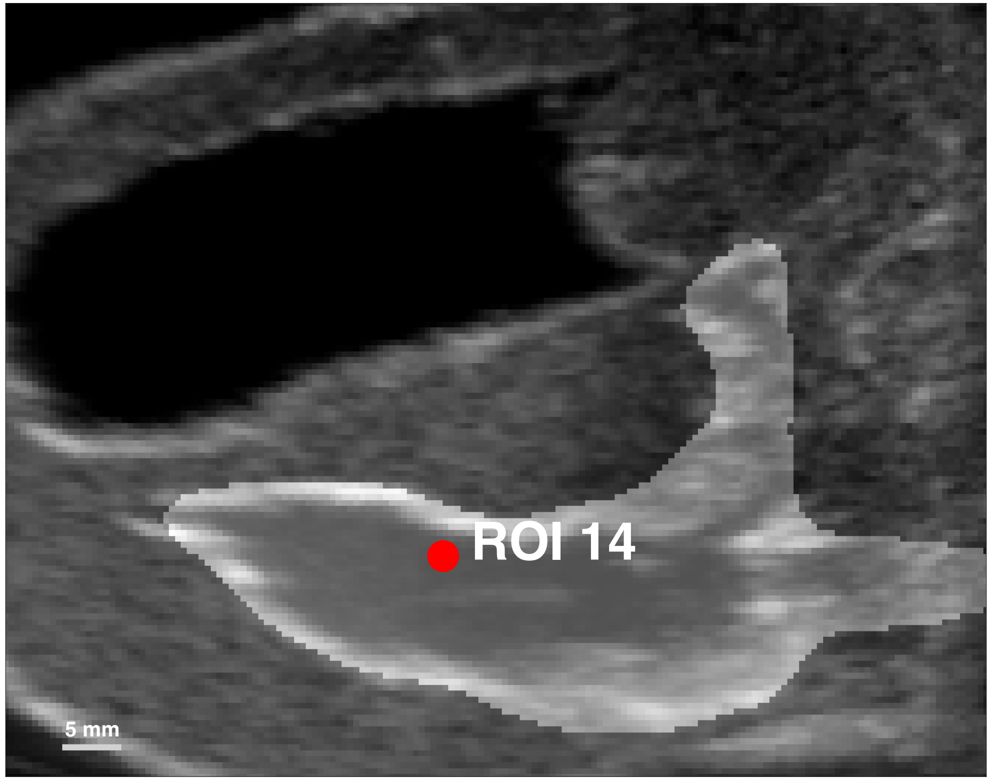

ROI #15

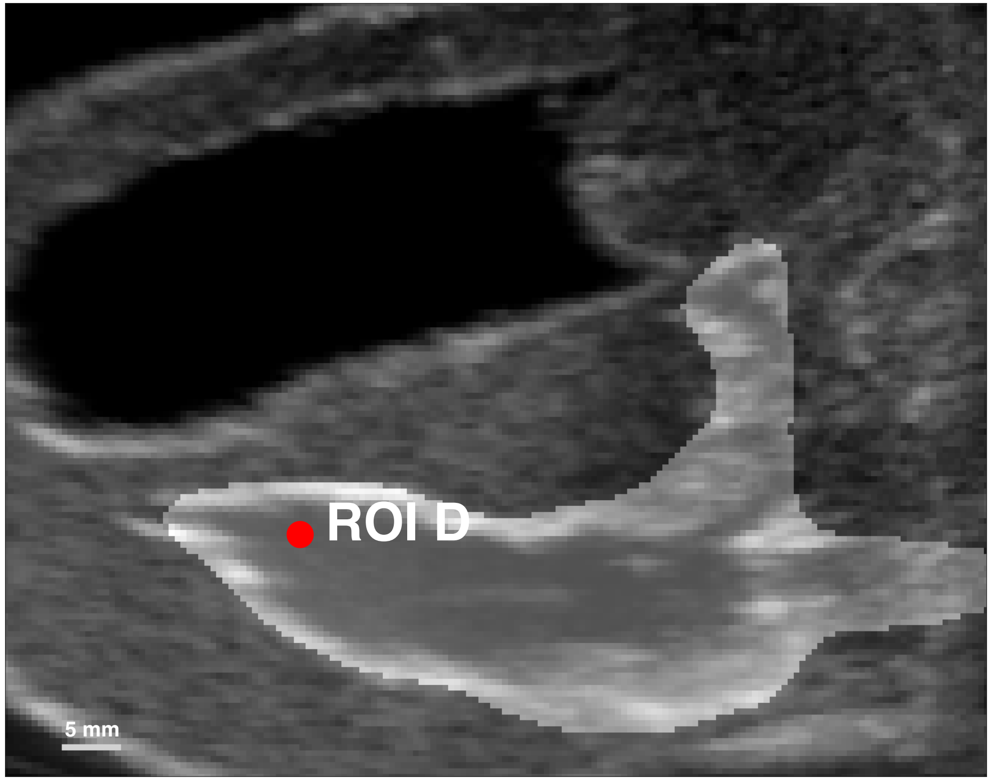

ROI #16

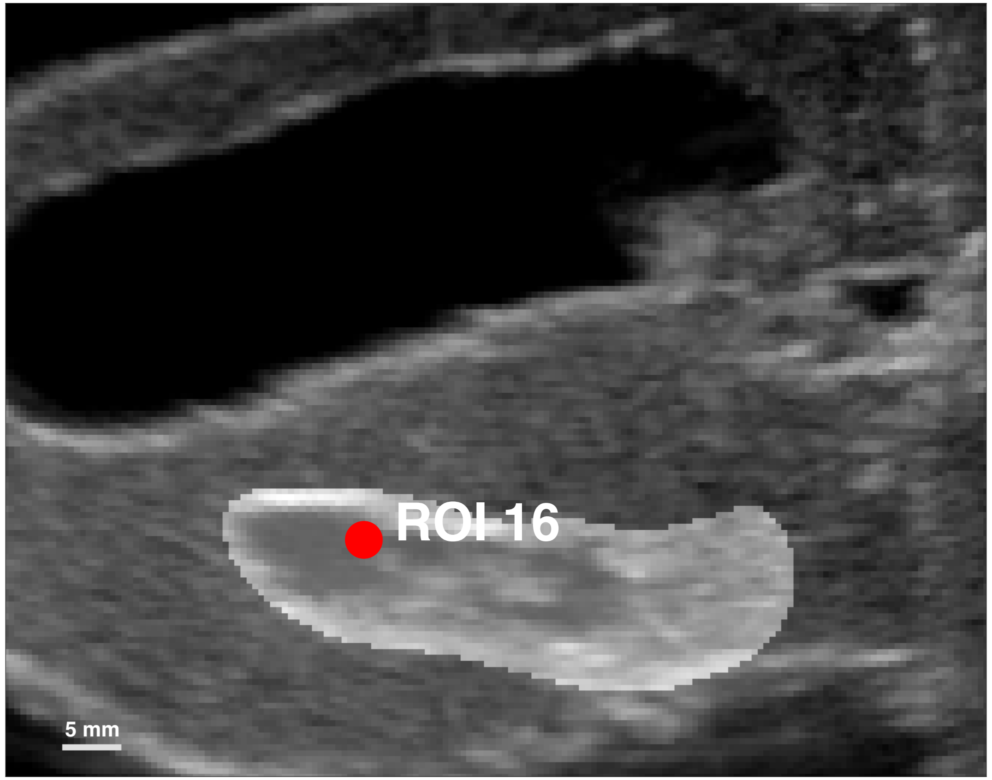

Supplement: Supplementary file 2 — Supplementary file 1 (pdf 1334 KB) [file 13239_2023_666_MOESM2_ESM.pdf]
