# Supplementary material for: Intra-Cardiac Flow from Geometry Prescribed Computational Fluid Dynamics: Comparison with Ultrasound Vector Flow Imaging
Source: Cardiovasc Eng Technol. 2023 Jun 15;14(4):489–504. doi: 10.1007/s13239-023-00666-2 (PMC10465406; doi:10.1007/s13239-023-00666-2)

Intra-Cardiac Flow from Geometry Prescribed Computational Fluid Dynamics: Comparison with Ultrasound Vector Flow Imaging

Journal: Cardiovascular Engineering and Technology

Authors: Rasmus Hvid[1], Matthias Bo Stuart[1], Jørgen Arendt Jensen[1] and Marie Sand Traberg[1*]

[1] Department of Health Technology, Technical University of Denmark, 2800 Kongens Lyngby, Denmark

*Corresponding author: Marie Sand Traberg, msene@dtu.dk

Evaluation of out-of-plane component

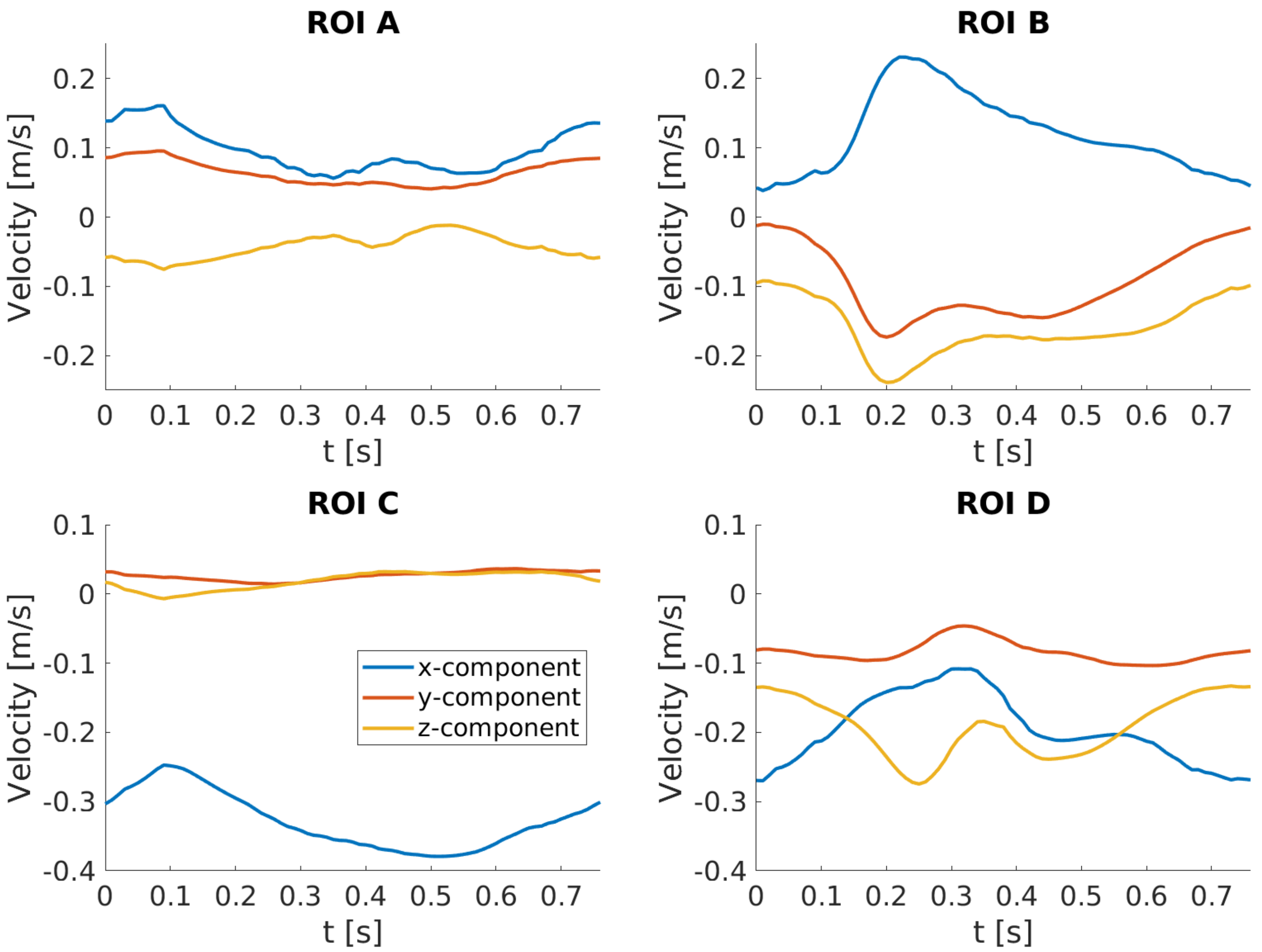

Supplement: Supplementary file 3 — Supplementary file 1 (pdf 161 KB) [file 13239_2023_666_MOESM3_ESM.pdf]
